# A Stability-Oriented Biomarker Selection Framework Synergistically Driven by Robust Rank Aggregation and L1-Sparse Modeling

**DOI:** 10.3390/metabo15120806

**Published:** 2025-12-18

**Authors:** Jigen Luo, Jianqiang Du, Jia He, Qiang Huang, Zixuan Liu, Gaoxiang Huang

**Affiliations:** 1 School of Intelligent Medicine and Information Engineering, Jiangxi University of Chinese Medicine, Nanchang 330004, China; 20192002@jxutcm.edu.cn (J.L.); 20201079@jxutcm.edu.cn (J.H.); 20201041@jxutcm.edu.cn (Q.H.); liuzixuan@jxutcm.edu.cn (Z.L.); huanggaoxiang@jxutcm.edu.cn (G.H.); 2 Key Laboratory of Traditional Chinese Medicine Artificial Intelligence of Jiangxi Province, Nanchang 330004, China; 3 School of Mathematics and Information Science, Nanchang Normal University, Nanchang 330032, China

**Keywords:** metabolomics, biomarker selection, feature selection stability, robust rank aggregation, L1-sparse modeling, stability-oriented feature selection framework

## Abstract

**Background**: In high-dimensional, small-sample omics studies such as metabolomics, feature selection not only determines the discriminative performance of classification models but also directly affects the reproducibility and translational value of candidate biomarkers. However, most existing methods primarily optimize classification accuracy and treat stability as a post hoc diagnostic, leading to considerable fluctuations in selected feature sets under different data splits or mild perturbations. **Methods**: To address this issue, this study proposes FRL-TSFS, a feature selection framework synergistically driven by filter-based Robust Rank Aggregation and L1-sparse modeling. Five complementary filter methods—variance thresholding, chi-square test, mutual information, ANOVA F test, and ReliefF—are first applied in parallel to score features, and Robust Rank Aggregation (RRA) is then used to obtain a consensus feature ranking that is less sensitive to the bias of any single scoring criterion. An L1-regularized logistic regression model is subsequently constructed on the candidate feature subset defined by the RRA ranking to achieve task-coupled sparse selection, thereby linking feature selection stability, feature compression, and classification performance. **Results**: FRL-TSFS was evaluated on six representative metabolomics and gene expression datasets under a mildly perturbed scenario induced by 10-fold cross-validation, and its performance was compared with multiple baselines using the Extended Kuncheva Index (EKI), Accuracy, and F1-score. The results show that RRA substantially improves ranking stability compared with conventional aggregation strategies without degrading classification performance, while the full FRL-TSFS framework consistently attains higher EKI values than the other feature selection schemes, markedly reduces the number of selected features to several tens of metabolites or genes, and maintains competitive classification performance. **Conclusions**: These findings indicate that FRL-TSFS can generate compact, reproducible, and interpretable biomarker panels, providing a practical analysis framework for stability-oriented feature selection and biomarker discovery in untargeted metabolomics.

## 1. Introduction

Feature selection is essential for building high-performance classification models in high-throughput bioinformatics studies like metabolomics and transcriptomics. It is also necessary to find potential biomarkers and facilitate subsequent biological interpretation and clinical translation [1]. Strong correlations between variables, a large percentage of noisy and redundant features, and a feature dimension that significantly surpasses the sample size are common characteristics of high-throughput omics data when compared to traditional low-dimensional data. Common feature selection techniques are quite sensitive to the selection of training-test splits, resampling techniques, and slight sample perturbations in these circumstances. Reduced reproducibility and translational value of the candidate biomarker panels can result from repeatedly applying the same approach on the same dataset, which can provide noticeably different feature subsets and poor stability of the chosen features [2]. According to earlier research, one of the main obstacles keeping omics-derived biomarkers from progressing from statistically significant findings to reliable, cross-cohort confirmed signatures is the volatility of feature selection outcomes.

Applying many filter-based feature selection techniques concurrently to screen differential metabolites is a popular tactic in practical metabolomics analysis. For instance, researchers frequently establish thresholds for fold change, the p-value of significance tests, and the variable relevance in projection obtained from partial least squares discriminant analysis. The candidate biomarker set is then determined by taking the intersection of these results [3,4,5]. Although such intersection-based strategies are intuitive and easy to implement, they suffer from at least two limitations. First, they usually provide only a binary “retain/remove” decision and ignore the continuous gradient of relative importance among features, making it difficult to distinguish metabolites that lie at different importance levels. Second, different filter methods rely on distinct statistics or model assumptions, yet the consistency and complementarity of their outputs are rarely quantified in a systematic way. As a result, it is difficult for researchers to judge whether the feature sets obtained by a given combination strategy are sufficiently stable and reliable under sample perturbations. In addition, extensive empirical and simulation studies have shown that many commonly used feature selection algorithms are extremely sensitive to the inclusion or exclusion of individual samples or to the way training sets are partitioned in high-dimensional, small-sample settings. Even when the classification performance remains high, the corresponding selected feature subsets can still differ considerably across runs [6,7,8].

From a methodological perspective, existing feature selection techniques are generally categorized into filter, wrapper, and embedded methods [9]. To mitigate the strong dependence of a single feature selector on specific sample splits and parameter choices, a variety of stability-oriented feature selection approaches have been proposed in recent years based on ensemble learning and perturbation ideas. These include heterogeneous ensembles based on function perturbation, stability selection schemes driven by sample perturbation, and hybrid frameworks that combine both types of perturbations [10,11,12]. Typically, such methods aggregate the outputs obtained from multiple resampled datasets or multiple base learners using mechanisms such as majority voting or rank aggregation, thereby reducing the randomness of single-run results and improving the robustness of feature selection to data perturbations in high-dimensional, small-sample scenarios.

However, most existing work still regards final classification performance as the primary optimization objective and treats stability as a secondary diagnostic index that is reported only in the results section, rather than promoting feature selection stability to a core design constraint on par with discriminative performance at the framework level [13,14]. On the one hand, many ensemble-based feature selection methods stop at the level of ranking or a single Top-K truncation, focusing on obtaining a more stable ranking list but lacking a systematic design for further compressing and optimizing the candidate feature space via sparse modeling under a given classification task. On the other hand, current studies differ considerably in terms of perturbation schemes, stability metrics, and baseline methods, and there is a lack of reproducible and comparable benchmarks for stability evaluation. This heterogeneity weakens the comparability and generalizability of stability-related conclusions across methods. In particular, for high-dimensional, small-sample settings such as metabolomics and gene expression, it remains an open problem how to substantially improve the consistency of selected features under resampling and perturbation while maintaining classification performance and how to link this consistency with the control of biomarker panel size and interpretability.

This paper presents a biomarker stability-oriented framework powered by Robust Rank Aggregation and L1-sparse modeling in order to overcome these limitations. It does this by adopting a “stability-first” approach that highlights the synergy between heterogeneous ensemble feature selection and sparse modeling. To calculate feature scores, five complementary filter techniques are first used in parallel: variance thresholding, chi-square test, mutual information, one-way ANOVA F test, and ReliefF. Robust Rank Aggregation is then used to merge their varied ranking lists in order to create a consensus feature ranking that is robust under many evaluation perspectives and to suppress the bias and instability of any particular scoring criterion at the methodological level. The candidate feature subsets created by keeping various top-ranked proportions of the RRA ranking are then used to construct an L1-regularized logistic regression model. This L1-sparse modeling module establishes a synergy between feature selection stability, feature compression, and classification performance by performing fine-grained screening and redundancy reduction within the candidate space by tightly coupling coefficient shrinkage with the target classification task. The suggested framework, called FRL-TSFS (filter-based Robust Rank Aggregation and L1-sparse modeling–driven feature selection framework), adheres to a “construct a robust candidate ranking and then perform task-aware sparse refinement within the candidate space” design as opposed to applying Lasso directly to the entire feature space or depending only on filter-based ensembles. Under high-dimensional, small-sample conditions, this integrated RRA–L1 method successfully reduces the susceptibility of L1-based models to noise and partition perturbations and makes it easier to find more compact and stable biomarker sets.

The main contributions of this study can be summarized as follows. First, from a stability-first viewpoint, we develop FRL-TSFS, a stability-oriented feature selection framework synergistically driven by Robust Rank Aggregation and L1-sparse modeling. By structurally integrating heterogeneous filter-based scoring with embedded sparse modeling, FRL-TSFS implements a hierarchical design that first constructs a robust candidate ranking and then performs task-aware sparse refinement within the candidate space, thereby elevating feature selection stability from a post hoc index to a core design principle on par with classification performance. Second, under a unified mildly perturbed scenario induced by 10-fold cross-validation, we introduce the Extended Kuncheva Index as a quantitative measure of feature subset consistency and establish a stability evaluation benchmark that covers different rank aggregation strategies and RRA-integrated feature selection frameworks. Through ablation studies, we show that, for high-dimensional omics data, the major gain in stability arises from introducing L1-sparse modeling within the pre-screened candidate space generated by heterogeneous ensemble filters, rather than from simply increasing the number of filter methods. Third, using six representative real-world datasets, we conduct a systematic comparison of FRL-TSFS and multiple baseline approaches from the three perspectives of stability, feature compression, and classification performance. The results demonstrate that FRL-TSFS can substantially improve feature selection stability and greatly reduce the number of selected biomarkers with minimal loss of discriminative performance. The resulting biomarker panels achieve a more balanced trade-off among size, reproducibility, and interpretability, providing a methodologically meaningful reference paradigm for biomarker discovery in untargeted metabolomics.

## 2. Related Work

Feature selection is a core component of high-dimensional, small-sample omics data analysis, and different method families exhibit markedly different behavior in terms of stability and interpretability. According to how they are coupled with the learning algorithm, existing feature selection methods are commonly grouped into three categories: filter, wrapper, and embedded approaches. Filter methods independently score individual features based on statistical tests, correlation measures, or information-theoretic criteria, such as analysis of variance, chi-square test, mutual information, and correlation coefficients. These methods have been widely used in metabolomics and gene expression studies because they are computationally efficient, easy to implement, and decoupled from specific classification models [15]. However, since they inherently ignore feature–feature interactions and redundancy structures, filter methods tend to be sensitive to the choice of training–test splits and sample perturbations when the feature dimension far exceeds the sample size and multicollinearity is pronounced. As a result, the overlap between selected feature sets across different resampling runs or cross-validation folds is often unsatisfactory [16]. Wrapper methods, in contrast, evaluate the quality of feature subsets by repeatedly training a classifier during the search over the subset space. Representative examples include recursive feature elimination and its combinations with support vector machines or ensemble tree models. Although wrapper approaches can improve predictive performance, they require searching over an extremely large candidate subset space and thus suffer from high computational cost and susceptibility to local optima in ultra–high-dimensional omics settings [17]. Embedded methods integrate feature selection into the model training process, such as L1-regularized linear models, least absolute shrinkage and selection operator (Lasso) and its variants, and tree-based ensemble methods. These approaches can strike a balance between predictive performance and feature compression to some extent, but their outputs are typically sensitive to model hyperparameters and sample splits, and their stability remains difficult to guarantee in high-dimensional, small-sample scenarios [18].

Stability-oriented feature selection techniques based on perturbation and ensemble concepts have garnered significant interest in recent years as a way to mitigate the substantial dependence of a single feature selector on particular sample partitions and parameter configurations [19]. From the perspective of the perturbed object, related studies can be broadly categorized into three classes: sample perturbation, feature/function perturbation, and hybrid perturbation combining both. Sample perturbation methods usually apply the same feature selection algorithm repeatedly on multiple training subsets generated by bootstrap resampling or repeated cross-validation and then summarize the results based on selection frequencies or the overlap of selected sets across subsets. This line of work has been systematically developed under the framework of stability selection [20]. A representative example is the subsampling-based Lasso stability selection framework proposed by Meinshausen and Bühlmann [11]. In this approach, Lasso models are repeatedly fitted on multiple random subsamples of the data, selection probabilities are computed for each feature, and features whose selection probabilities exceed a predefined threshold are retained. This framework provides attractive theoretical guarantees on error control and has been widely validated in various biomedical applications. However, in ultra-high-dimensional, small-sample omics settings, stability selection typically requires a large number of subsampling and model-fitting steps, which leads to substantial computational cost; at the same time, because each fit uses only a fraction of all available samples, the already limited sample size is further reduced, potentially resulting in less stable parameter estimation. Feature or function perturbation methods, on the other hand, apply multiple heterogeneous feature selection algorithms in parallel on the same dataset or repeatedly run the same algorithm under different initializations or hyperparameter settings. Their outputs are then integrated using majority voting, score averaging, Borda count, Robust Rank Aggregation, or other rank aggregation strategies to mitigate the bias and randomness of any single method [21]. Some studies further combine sample and function perturbations to construct hybrid stability frameworks, in which multiple resampling schemes and multiple feature selectors are jointly perturbed to obtain feature subsets that are robust under a broader spectrum of perturbation conditions [22]. Overall, these methods alleviate, to some extent, the sensitivity of feature selection results to data perturbations and model specifications under high-dimensional, small-sample conditions, but they mostly aim at obtaining a more stable feature ranking or Top-K candidate set. They pay relatively little attention to how sparse modeling can be further combined, for a given classification task, to perform task-coupled optimization and compression of the candidate space, which is precisely the problem that the present study seeks to address.

Numerous studies have investigated the use of rank aggregation and ensemble feature selection for biomarker development in metabolomics and other omics applications [23]. On the one hand, it is common practice to score metabolites or genes using multiple filter methods (e.g., *t*-test, ANOVA, mutual information, and correlation analysis) and then aggregate these rankings via arithmetic mean ranks (Mean), Borda count, or Robust Rank Aggregation (RRA) to identify “consensus features” that consistently appear near the top under different scoring criteria. Existing evidence suggests that, compared with single-filter methods, rank aggregation strategies can moderately improve the consistency of feature selection results under resampling conditions without noticeably compromising classification performance [24]. On the other hand, some studies use rank aggregation to construct a stable candidate feature set and then train downstream classifiers or regression models on this set for tasks such as disease subtyping and prognosis prediction [25]. Nevertheless, in most of these works, rank aggregation is treated as an independent preprocessing step—namely, “first obtain a relatively stable ranking or Top-K candidate subset via ensemble methods, then train a classification model on this subset”—and the potential synergy between rank aggregation and subsequent embedded sparse modeling is rarely examined systematically. For example, existing research typically does not disentangle whether stability gains primarily stem from the rank aggregation itself or from the sparse modeling performed within the candidate space, and few studies conduct comprehensive comparisons of different ways of combining rank aggregation with embedded modeling in terms of stability and feature compression under a unified perturbation setting.

From the viewpoint of evaluation, current studies on feature selection stability also exhibit substantial heterogeneity in perturbation schemes, stability metrics, and baseline methods [26]. Some works adopt the Jaccard index or Kuncheva index to quantify the overlap between feature subsets obtained from different runs, while others extend these indices to accommodate cross-validation and multi-class classification scenarios [19]. However, due to differences in perturbation strength (e.g., number of folds, resampling ratios), stability metrics (e.g., whether ranking information is considered), and the choice of comparison methods, conclusions drawn across studies are often not directly comparable. In application domains such as metabolomics, there is still a lack of reproducible stability evaluation benchmarks that support meaningful cross-study comparison. In particular, for high-dimensional, small-sample metabolomics data, an important yet relatively under-explored question is how to systematically compare different rank aggregation strategies and their combinations with embedded modeling approaches in terms of their joint performance on stability, feature compression, and classification accuracy under a unified perturbation scenario, and how to analyze the main sources of stability gains.

Against this backdrop, current research on feature selection stability still reveals clear gaps at both the methodological and evaluation levels. On the one hand, most studies treat rank aggregation as an independent preprocessing step and stop at obtaining a more stable ranking or Top-K candidate set, without systematically investigating the interaction between rank aggregation and subsequent embedded sparse modeling or comparing different ways of combining rank aggregation with embedded modeling in terms of stability and feature compression. On the other hand, substantial variability in perturbation schemes, stability metrics, and baseline configurations has hindered the establishment of reproducible, cross-comparable stability benchmarks in specific application domains such as metabolomics. Motivated by these gaps, the present study focuses on stable biomarker selection in metabolomics and gene expression data from the perspective of synergistic heterogeneous ensemble feature selection and L1-sparse modeling. Adopting a “stability-first” design principle, this work constructs a unified perturbation scenario based on 10-fold cross-validation together with an evaluation system centered on the Extended Kuncheva Index, systematically compares different rank aggregation strategies and their combinations with embedded modeling approaches in terms of their joint performance on stability, feature compression, and classification, and, on this basis, proposes FRL-TSFS, a feature selection framework synergistically driven by Robust Rank Aggregation and L1-sparse modeling.

## 3. Data and Methods

### 3.1. Datasets

#### 3.1.1. Benchmark Datasets

This work comprised six representative datasets, including public benchmark data, animal models, and human cohorts, in order to thoroughly assess the behavior of various feature selection algorithms across different metabolomics data settings (Table 1). These datasets differ in sample size, feature dimensionality, and class structure, and all exhibit the typical high-dimensional, small-sample property with *p* ≫ *n*, making them suitable for systematically assessing the stability, feature compression capability, and classification performance of feature selection methods in metabolomics and gene expression settings.

(1) Adenocarcinoma lung cancer study (human). This dataset was generated for biomarker discovery in plasma metabolomics of adenocarcinoma lung cancer. It comprises 181 plasma samples analyzed by gas chromatography–mass spectrometry (GC–MS), yielding abundance profiles for 511 metabolites. The dataset and details of the experimental design are available from the Metabolomics Workbench under study ID ST000385.

(2) Asthma/obesity-related study (mouse). This dataset investigates the impact of different diets on the arginine metabolic pathway in mouse lung tissue. It contains 80 lung tissue samples, which were profiled using GC-MS, resulting in abundance measurements for 614 metabolites. The dataset can be accessed from the Metabolomics Workbench under study ID ST000419.

(3) GQDL intervention in prediabetic rats (GQDL). This dataset was provided by the Research Center for the Differentiation and Development of Basic Theory of Traditional Chinese Medicine at Jiangxi University of Chinese Medicine. A high-fat diet was used to induce an insulin resistance model mimicking prediabetes in rats, followed by intervention with the traditional Chinese medicine formula Gegen Qinlian Decoction (GQDL). Adipose tissue samples were collected and analyzed using lipidomics techniques to evaluate the effects of GQDL on lipid metabolic profiles and diabetes-related biochemical indicators.

(4) Shenfu injection treatment for cardiogenic shock (SFCS). This dataset was also provided by the same research center at Jiangxi University of Chinese Medicine. A rat model of intermediate-stage cardiogenic shock was established by ligating the left anterior descending coronary artery near the cardiac apex. Different doses of Shenfu injection were then administered as intervention. Biological samples were collected 60 min after administration, and multiple pharmacodynamic indices were measured to assess the metabolic effects of Shenfu injection. In the present study, the blank, model, and treatment groups were used as the analysis cohorts.

(5) Leukemia (public gene expression data). The Leukemia dataset was originally introduced by Golub et al. [27] to investigate gene expression differences between acute lymphoblastic leukemia (ALL) and acute myeloid leukemia (AML). It contains 72 samples in total (47 ALL and 25 AML) with 7129 gene expression features. This dataset has been widely used as a benchmark for evaluating feature selection methods in high-dimensional, small-sample scenarios.

(6) Arcene (NIPS 2003 Feature Selection Challenge). The Arcene dataset was released as part of the NIPS 2003 Feature Selection Challenge for a binary classification task distinguishing cancer patients from normal controls. It consists of 100 training samples and 100 test samples, with 10,000 features in total. Only a subset of these features is truly informative, while the remaining variables are artificially added noise. This design makes Arcene a challenging benchmark for assessing the robustness of feature selection methods in the presence of extensive redundancy and noise.

From a methodological perspective, FRL-TSFS does not impose a strict analytical lower bound on the number of samples per class. However, in practice the usable sample size is constrained by the requirements of the statistical filters and the embedded models. In particular, the chi-square test, one-way ANOVA, and L1-regularized logistic regression all require a certain number of observations in each class to yield stable estimates. In this study, we adopted stratified 10-fold cross-validation, which implicitly requires that each class be represented by at least 10 samples so that every fold contains at least one observation from each class. In our benchmark datasets, the per-class sample sizes are all above this threshold (Table 1), and within this range the FRL-TSFS pipeline behaved numerically stable.

#### 3.1.2. Data Preprocessing and Normalization

Before feature selection, all datasets were subjected to a unified preprocessing and normalization pipeline. For the four metabolomics datasets (GQDL, SFCS, ST000385, and ST000419), missing values were imputed using feature-wise means, and metabolite intensities were log-transformed (where necessary) to reduce right-skewness. The log-transformed intensities were then normalized by level scaling, i.e., dividing each feature by its mean across samples, in order to mitigate between-feature magnitude differences. For the two gene expression datasets (Arcene and Leukemia), we started from the preprocessed expression matrices provided by the original sources and applied the same feature-wise level scaling, so that all six omics datasets entered the FRL-TSFS pipeline on a comparable normalized scale.

To justify the choice of normalization scheme, we empirically compared several commonly used approaches for high-dimensional omics data, including AutoScaling (mean-centering followed by division by the standard deviation), variance stabilizing normalization (VSN), simple log-transformation, level scaling, and cyclic loess normalization. For each dataset and each normalization method, we quantified within-group variability using three measures: the pooled coefficient of variation (PCV), the pooled error variance (PEV), and the pooled median absolute deviation (PMAD), where a lower PMAD indicates more effective removal of experimental noise. As summarized in Supplementary Table S1, level scaling consistently yielded the lowest or near-lowest PMAD values across all six datasets, while avoiding the numerical instability or excessive rescaling observed for some of the other methods (e.g., cyclic loess or VSN). On this basis, level scaling was adopted as the default normalization strategy for all six metabolomics and gene expression datasets in the main analysis. We did not rely on housekeeping-gene–based normalization, because the stability of candidate housekeeping genes is not guaranteed across the heterogeneous public datasets considered here, and global feature-wise scaling is more appropriate for ensuring comparability of predictors in this context.

### 3.2. A Feature Selection Framework Synergistically Driven by Filter-Based Robust Rank Aggregation and L1-Sparse Modeling, FRL-TSFS

In high-dimensional, small-sample omics settings, previous studies have shown that many commonly used feature selection approaches, including L1-regularized embedded methods, may suffer from instability and yield molecular signatures whose sparsity, predictive performance, and reproducibility are difficult to balance simultaneously [20]. On the one hand, the extremely large number of features relative to the limited number of samples amplifies the effects of feature correlations and noisy variables, making the solution of the least absolute shrinkage and selection operator (Lasso) highly sensitive to training–test splits and mild sample perturbations. As a result, the selected feature sets can fluctuate considerably across repeated experiments. On the other hand, although single-filter methods or simple rank aggregation can to some extent improve the robustness of the initial ranking, they typically stop at the level of ranking or a fixed Top-K truncation and do not fully exploit discriminative information that is directly tied to the classification task.

**Figure 1:**
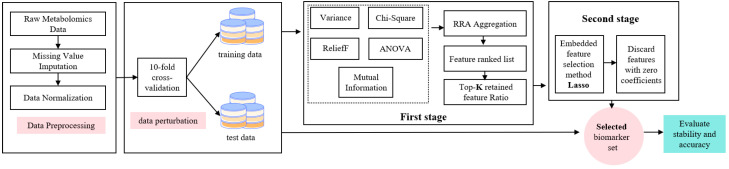
A feature selection framework synergistically driven by filter-based RRA and L1-sparse modeling.

Motivated by these considerations, this study proposes FRL-TSFS, a feature selection framework synergistically driven by filter-based RRA and L1-sparse modeling. In this framework, multiple filter-based scoring functions are first combined via RRA to construct a candidate feature ranking that is less sensitive to the bias of any single scoring criterion. Then, L1-regularized logistic regression is applied to the candidate subset to perform task-aware sparse refinement, thereby establishing a synergy among feature selection stability, feature compression capability, and classification performance. The overall workflow of FRL-TSFS is illustrated in Figure 1:

#### 3.2.1. Heterogeneous Filter-Based Feature Ranking via Robust Rank Aggregation

Given that metabolomics data may simultaneously exhibit linear and non-linear relationships, continuous and discrete variables, as well as local neighborhood structures, the first stage of the proposed framework adopts five complementary filter methods—variance thresholding, chi-square test, mutual information (MI), one-way ANOVA F test, and ReliefF—to construct a heterogeneous set of feature scores. Variance thresholding removes near-constant or low-information features from the perspective of overall variability. The chi-square test is suitable for characterizing the association between discrete features and class labels. Mutual information measures the reduction in uncertainty of the class label given a feature and, unlike Pearson correlation, is sensitive to arbitrary (including non-linear) forms of statistical dependence between them [28]. The ANOVA F test quantifies the discriminative power of continuous features across classes by comparing between-group and within-group variance. ReliefF, in turn, evaluates the contribution of each feature to class boundaries based on local neighborhood information in the sample space.

In all cases, each filter assigns scores to the full set of candidate features (after removing constant or near-zero-variance variables) and returns a complete ranking list, rather than a method-specific truncated subset. Consequently, the dimensionality of the feature space entering the subsequent RRA step is identical across all five filters, and the number of features finally passed to the embedded L1 model is controlled by the predefined Top-K cut-offs applied to the RRA consensus ranking rather than by any individual filter. By applying these five scoring functions in parallel on the same dataset and subsequently aggregating their rankings using RRA, the framework integrates the strengths of different evaluation paradigms and mitigates the bias and instability of any single filtering criterion, thereby providing a more robust candidate feature ranking for the downstream L1-sparse modeling stage.

RRA is a robust rank-based aggregation method that integrates multiple candidate ranking lists to obtain a globally consistent consensus ranking [29]. After generating feature rankings using the five filter methods described above, this study applies RRA to aggregate these heterogeneous ranking lists and construct a candidate feature ranking that is less sensitive to the bias of any single scoring criterion under function perturbation conditions. Suppose there are *L* filter–style feature selection methods, and each method assigns a ranking from 1 to *N* over all *N* features on the training set. Let $r_{i,j}$ denote the rank of feature *i* in the *j*-th list (i=1,…,N;j=1,…,L). To make rankings from different lists comparable, we normalize each rank as

(1) $$p_{i,j}=\frac{r_{i,j}}{N}\;\;i=1,\ldots ,N;j=1,\ldots ,L$$

Under the null hypothesis of random ranking, the normalized ranks $p_{i,j}$ are independent and identically distributed (i.i.d.) as U(0,1) for each feature *i*. Let $p_{i(1)}\leq p_{i(2)}\leq \ldots \leq p_{i(L)}$ be the order statistics of $\left\{p_{i,1},\ldots ,p_{i,L}\right\}$, where $p_{i(s)}$ is the *s*-th smallest normalized rank. For a given s the cumulative distribution of $p_{i(s)}$ under the null is

(2) $$P(P_{\left(s\right)}\leq x)=\sum_{j=s}^{L}\left(\begin{array}{l} L\\ j \end{array}\right)x^{j}{\left(1-x\right)}^{L-j},0\leq x\leq 1$$

This is the probability that at least *s* of the *L* normalized ranks are no greater than *x*. RRA then defines an aggregate significance score for feature *i* as

(3) $$\rho_{i}=\underset{s=1,\ldots ,L}{\min}\{P(P_{\left(s\right)}\leq p_{i(s)})\}$$

A smaller $\rho_{i}$ indicates that feature *i* tends to appear nearer the top across multiple ranking lists and is therefore more important. In practice, $\rho_{i}$ values are adjusted for multiple testing (e.g., Benjamini–Hochberg FDR correction), and the adjusted *p*-values can be transformed (e.g., −*log*_10_) to produce a final consensus score and a stable global ranking of all features.

Leveraging its strong resistance to outliers and clear statistical interpretation, RRA has been widely used to integrate results from multiple models or pipelines and to reduce the bias introduced by any single ranking method, thereby improving the stability and reliability of feature selection outcomes. In our workflow, after obtaining the RRA consensus ranking, we select the top-K proportion of features to form candidate biomarker sets, which are then passed to the Lasso-based sparse modeling module for further fine-grained screening.

#### 3.2.2. Lasso-Based Sparse Modeling and Fine-Grained Screening

After the RRA-based rank aggregation step, we consider several predefined Top-K retention ratios between 20% and 50% (20%, 25%, 30%, 35%, 40%, 45%, and 50%) to form candidate subsets of different sizes for the subsequent embedded sparse modeling. This range covers relatively aggressive compression (around 20%) to moderately conservative retention (around 50%) and, in our datasets, typically corresponds to tens to low hundreds of metabolites or genes, which is consistent with the typical scale of biomarker panels reported in previous omics studies. It therefore allows us to systematically examine the trade-off between feature selection stability, predictive performance, and feature compression under a realistic range of candidate-set sizes. Features whose ranks fall within the corresponding Top-K percentile of the RRA consensus ranking are then passed to the L1-regularized logistic regression model for second-stage sparse selection and stability evaluation.

Within each candidate space, an L1-regularized logistic regression model (Lasso) is then fitted to perform task-aware sparse refinement [30]. Compared with directly fitting Lasso on the full feature space, conducting L1-sparse modeling on a prefiltered candidate set obtained from heterogeneous filter methods helps, on the one hand, to alleviate the interference of noisy features and strong multicollinearity on the interpretation pathway under high-dimensional, small-sample conditions. On the other hand, because the Lasso objective combines a classification loss term with an L1 regularization term, its sparsity-inducing penalty compresses redundant features while retaining those that are highly relevant to the classification task, thereby creating a coupling between “robust candidate ranking” and “task-aware sparse refinement”.

To formalize this process, consider a given fold of cross-validation, where the metabolomics data in the training set are represented by a feature matrix $X\in {\mathbb{R}}^{m\times n}$ and a response vector $Y\in {\mathbb{R}}^{m}$, with *m* samples and *n* metabolites. For a binary classification task, the Lasso objective can be written as

(4) $$\underset{\beta }{min}L(\beta ;X,y)+\lambda \;{∥\;\beta \;∥}_{1}$$

where $L\left(\beta ;X,y\right)$ denotes a classification loss based on the log-likelihood (e.g., cross-entropy loss), and *λ* > 0 is the regularization parameter controlling the degree of sparsity. The objective in (4) consists of a data-fitting loss term and an L1 regularization term. The latter penalizes the absolute values of the regression coefficients and drives a subset of them exactly to zero, yielding a sparse solution. For high-dimensional, small-sample data that are common in bioinformatics and medical studies, where features often exhibit strong multicollinearity, Lasso leverages L1 regularization to shrink redundant variables within correlated feature groups. This not only mitigates the adverse impact of multicollinearity on feature selection results but also enhances the interpretability of the resulting models.

For multiclass metabolomics classification tasks, this study adopts a one-vs-rest strategy to decompose the multiclass problem into several binary sub-tasks. For each class *c* (class index), an indicator label $z_{i}^{\left(c\right)}=I\left(y_{i}=c\right)$ (indicator label for class *c*) is constructed, and an L1-regularized logistic regression model of the form in (4) is fitted on $\left(X,z^{\left(c\right)}\right)$. This yields a class-specific coefficient vector $\beta^{\left(c\right)}$ (coefficient vector for class *c*). After model training, the coefficient vectors from all classes are summarized (for example, by taking the mean of the absolute coefficients across classes) to obtain an aggregated coefficient vector $\bar{\beta }$ (aggregated coefficient vector). A set of features is then selected according to the magnitudes of $\bar{\beta }$ (absolute coefficient values), and those with the largest absolute coefficients constitute the final feature subset.

To reduce the impact of manually chosen regularization strength on the results, the regularization parameter *λ* (regularization parameter) for Lasso is automatically tuned by cross-validation within each candidate feature set. In addition, Lasso models are trained independently on different Top-K candidate feature subsets, and the above coefficient-based shrinkage procedure is applied to each of them. This design enables a systematic comparison of different RRA-integrated feature selection frameworks in terms of feature selection stability and classification performance. Benefiting from the sparsity-inducing property of L1 regularization, the Lasso-based refinement step can substantially reduce the number of selected features while preserving classification performance, thereby enhancing the interpretability of the final results.

It is worth noting that in FRL-TSFS only the filter scores are converted into ranks for the purpose of aggregation in the RRA module. After RRA produces a consensus ranking, the Top-K% features are taken as a candidate subset, and a multiclass L1-regularized logistic regression model is fitted on the normalized measurement values of these candidate features to perform sparse selection. The features with non-zero coefficients are then used to construct the final panel. A linear SVM classifier is subsequently trained on the normalized measurement values of the L1-selected features and evaluated on the test data. In other words, FRL-TSFS uses ranking only as an intermediate representation for feature selection, while both the embedded L1 model and the final SVM classifier operate in the original measurement-based feature space with reduced dimensionality.

### 3.3. Evaluation Metrics

To comprehensively assess the performance of the proposed FRL-TSFS framework on metabolomics data, we evaluate it from two perspectives: the stability of the selected feature sets and the classification performance.

(1) Stability metric.

Since feature selection stability is the core design objective of the FRL-TSFS framework, the choice of stability metric is particularly critical. In conventional studies, the Jaccard index or the Kuncheva index is often used to quantify the overlap between feature subsets obtained from different runs. However, under cross-validation and repeated resampling scenarios, the original Kuncheva index is quite sensitive to the total number of features and to the sizes of the selected subsets. Therefore, this study adopts the Extended Kuncheva Index (EKI) as the main quantitative measure of feature selection stability and uses it to evaluate the consistency of feature subsets obtained across multiple cross-validation folds [31].

(5) $$EKI=\frac{2}{W(W-1)}\sum_{i=1}^{W-1}\sum_{j=i+1}^{W}\frac{\left|S_{i}\cap S_{j}\right|-\frac{\left|S_{i}\right|*|S_{j}|}{n}}{\min(\left|S_{i}\right|,\left|S_{j}\right|)-\max(0,\left|S_{i}\right|+\left|S_{j}\right|-n)}$$

where $S_{i}$ and $S_{j}$ denote the feature subsets selected, respectively, *W* is the number of cross-validation repetitions, and *n* is the total number of candidate features. The value of *EKI* typically lies in the range [−1, 1]. Values close to 1 indicate that the selected feature subsets are highly consistent across different partitions; values close to 0 correspond to a level of agreement similar to random selection; and negative values suggest a tendency for mutual exclusion between subsets. In this study, under the mildly perturbed scenario induced by 10-fold cross-validation, the mean and standard deviation of *EKI* are used as the primary indicators for assessing the stability of different feature selection methods.

(2) Metrics for Classification Performance

An essential part of metabolomics research is automatic sample identification, which is commonly implemented via classification models built on metabolite profiles. In this study, a linear Support Vector Machine (SVM) is used as a unified downstream classifier to evaluate the quality of the feature subsets produced by different selection strategies: for each method, an SVM is trained on the selected features and its performance is assessed on the corresponding test sets. Two widely used metrics, Accuracy and F1-score, are adopted to ensure a fair and robust comparison. The formulas are as follows:

(6) $$Accuracy=\frac{TP+TN}{TP+TN+FP+FN}$$

*Accuracy* represents the proportion of correctly predicted samples in the test set, reflecting the overall classification performance. Here, *TP*, *TN*, *FP*, *FN* denote the numbers of true positives, true negatives, false positives, and false negatives, respectively.

The F1-score is the harmonic mean of *Precision* and *Recall*, and is particularly suitable for scenarios with imbalanced class distributions. It is calculated as follows:

(7) $$F1\text{-}score=\frac{2\cdot Precision\cdot Recall}{Precision+Recall}$$

In this study, 10-fold cross-validation (10-fold CV) is employed to evaluate the performance of the classifier. The average values of Accuracy and F1-score across all folds are reported as the final performance metrics.

### 3.4. Experimental Setup

To systematically evaluate the stability, classification performance, and feature compression capability of the proposed FRL-TSFS framework, a unified mildly perturbed scenario was constructed on each dataset using a 10-fold cross-validation strategy. This setting allows the generalization ability of the models to be examined under multiple training–test splits, while simultaneously introducing a mild perturbation of approximately 10% of the samples in each round by holding out about 10% of the data as the test set. In this way, the sensitivity of feature selection results to sample-level perturbations can be quantitatively assessed. The detailed procedure is as follows:

(1) The original dataset is randomly and evenly partitioned into 10 mutually disjoint subsets $\left\{D_{1},D_{2},D_{3},\ldots ,D_{10}\right\}$, each containing approximately 10% of all samples.

(2) In the *k*-th fold of cross-validation, subset $D_{k}$ is used as the test set, and the union of the remaining nine subsets, $U_{j\neq k}D_{j}$, is used as the training set to construct the *k*-th train–test split. By successively switching the test subset across the 10 folds, a series of training sets is obtained that are mutually independent but differ from each other by only about 10% of the samples, thereby simulating a mildly perturbed sample-level scenario.

(3) For each training set, the five filter-based feature selection methods—variance thresholding, chi-square test, mutual information, ANOVA F test, and ReliefF—are first applied independently in the original feature space to compute an importance score for each metabolite (or gene) and to generate the corresponding feature ranking lists.

(4) The five ranking lists are then fed into the RRA module for robust rank aggregation to obtain a unified consensus feature ranking. According to this aggregated ranking, the top 20%, 25%, 30%, 35%, 40%, 45%, and 50% of features are successively selected to construct candidate biomarker sets of different sizes for the subsequent embedded sparse modeling.

(5) For each candidate set, a multiclass Lasso-based logistic regression model is fitted on the current training set to perform sparse modeling and fine-grained feature screening. The non-zero coefficients are then used to define the selected feature subset. A linear SVM classifier is subsequently trained on the training samples restricted to this subset and evaluated on the corresponding test set, and the resulting classification Accuracy, F1-score, and other performance metrics are recorded.

(6) After completing 10-fold cross-validation, the 10 feature subsets obtained for each feature selection framework are treated as the objects for stability analysis, and the EKI is computed to quantify the similarity among feature subsets under different sample partitions.

Under this experimental setup, for each dataset, each feature selection method, and each candidate feature proportion, a pair of stability metrics (EKI values) and classification performance metrics (Accuracy and F1-score) is obtained. This enables a systematic comparison of FRL-TSFS and the baseline methods in terms of feature selection stability, classification performance, and feature compression capability within a unified “10% data perturbation” scenario.

To further assess the biological relevance of the feature panels produced by FRL-TSFS, we additionally performed a univariate differential analysis on all six datasets. For each feature (metabolite or gene), normalized abundances or expression levels were tested for group differences on the full dataset using Welch’s *t*-test for binary classification problems or one-way ANOVA for multi-class problems, followed by Benjamini–Hochberg false discovery rate (FDR) correction. Features with FDR-adjusted *p*-values < 0.05 were regarded as significantly differentially expressed/abundant (DE features). For each dataset, we then quantified the overlap between the FRL-TSFS panel (under the default Top-K and C settings) and the set of DE features, and calculated the proportion of panel members that were also identified as DE features. The detailed statistics of this overlap are summarized in Table 3.

## 4. Experiments and Results

To verify the effectiveness of the proposed FRL-TSFS framework for stability-oriented biomarker selection in high-dimensional omics data, systematic comparative experiments were conducted on the six metabolomics and gene expression datasets described above. The performance of different rank aggregation strategies and feature selection frameworks was comprehensively analyzed in terms of feature selection stability, classification performance, and feature compression capability. The main experimental design comprises three levels. First (Section 4.1), under a setting where only the rank aggregation strategy in the filter-ensemble component is varied, Robust Rank Aggregation (RRA) is compared with traditional aggregation methods such as Mean, Borda, and Intersection, in order to assess the impact of different rank aggregation schemes on feature selection stability and classification accuracy. Second (Section 4.2), with RRA fixed as the rank aggregation method, several embedded and wrapper algorithms—including L1-regularized logistic regression, gradient boosting decision tree (GBDT), Random Forest, recursive feature elimination with cross-validation (RFECV), and XGBoost—are coupled with RRA to construct different synergistic RRA-based feature selection frameworks, thereby systematically examining the overall advantages of FRL-TSFS in terms of stability, feature compression, and classification performance. Third (Section 4.3), ablation experiments are performed by removing the RRA-based filter ensemble and retaining only the embedded feature selector, so as to comparatively analyze the relative contributions of heterogeneous filter integration and L1-sparse modeling to the improvement in stability. In addition, Section 4.4 presents a sensitivity analysis of the penalty parameter C in the L1-regularized logistic regression, and Section 4.5 compares FRL-TSFS with a subsampling-based Lasso stability selection baseline (SS-Lasso) under the same evaluation protocol to further position the proposed framework among existing stability-oriented methods.

### 4.1. Comparison of Rank Aggregation Strategies Under a Heterogeneous Filter-Based Ensemble

**Figure 2:**
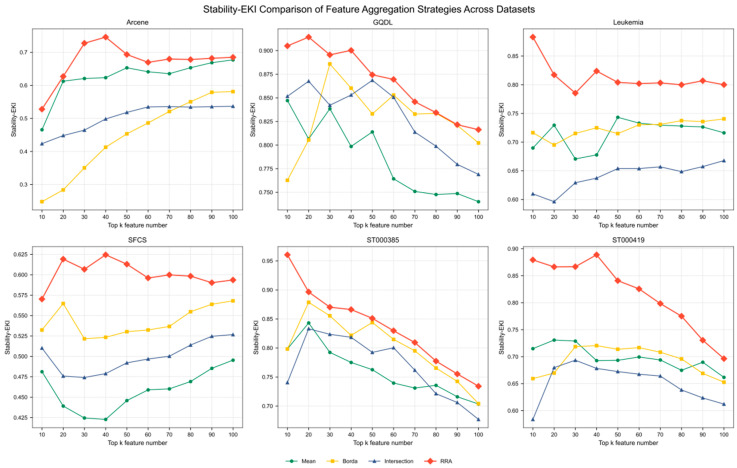
Stability–EKI comparison of feature aggregation strategies across six benchmark datasets. The four metabolomics datasets (GQDL, SFCS, ST000385, ST000419) have metabolite intensity features, whereas Arcene and Leukemia are gene expression datasets.

Based on the above experimental setup, we next compare different rank aggregation strategies from the perspectives of feature selection stability and classification performance. First, in conjunction with Figure 2, we analyze, within the unified heterogeneous filter-based ensemble framework, how the EKI values of each aggregation method vary across different datasets and Top-K settings (Top 10–Top 100 with a step size of 10), in order to evaluate their ability to maintain consistent feature subsets under sample perturbations.

In terms of stability, RRA shows a clear overall advantage over the other three aggregation strategies, including the arithmetic mean, across all six omics datasets. Specifically, on the ST000419, Leukemia, and SFCS datasets, the RRA-based aggregation strategy achieves the highest stability for all Top-K settings, with mean EKI values of 0.8167, 0.8125, and 0.6011, respectively. These correspond to relative improvements of 11.08%, 8.85%, and 5.84% over the second-best strategy. In contrast, the gains on the GQDL, ST000385, and Arcene datasets are slightly smaller, but RRA still remains superior, with mean improvements in the range of approximately 3.30–4.65%, highlighting its robustness under different data distributions.

Overall, RRA is either the best or tied for the best method across all Top-K intervals on all datasets, with an average EKI of 0.7674, which is markedly higher than that of Mean (0.6735), Borda (0.6728), and Intersection (0.6486). It is worth noting that the relative advantage of RRA gradually narrows as the size of the RRA-retained candidate biomarker set increases, but it still maintains a stable and consistently positive margin. This indicates that, under small-sample, high-dimensional conditions with a substantial proportion of noisy features, RRA can effectively mitigate the ranking bias of individual methods and significantly enhance the consistency of selected feature subsets across multiple sample partitions.

To further compare the classification performance of different rank aggregation strategies on each dataset, the classification Accuracy and F1-score of each method were recorded under different numbers of selected features. The results were then averaged over the 10 folds of 10-fold cross-validation and used as the performance indicators. The summarized results are reported in Supplementary Information Tables S2 and S3, which list the Accuracy and F1-score achieved by each aggregation strategy on each dataset, thereby characterizing their discriminative ability within a unified candidate feature space. On this basis, the statistical significance of the performance differences among the aggregation strategies will be further examined in conjunction with the subsequent significance analysis.

**Figure 3:**
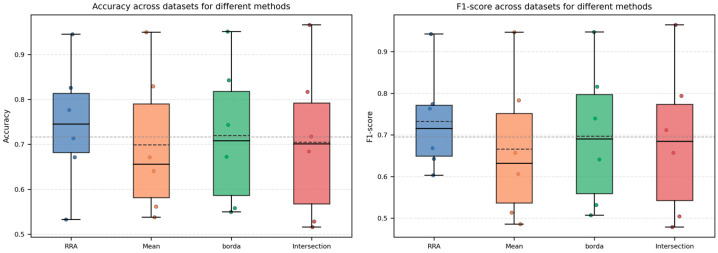
Boxplots of Accuracy and F1-score for different aggregation strategies.

Based on the classification results summarized in Supplementary Tables S2 and S3, boxplots of Accuracy and F1-score (Figure 3) were generated for the four aggregation strategies, with the individual dataset-specific values overlaid as scatter points. The plots show that the overall distributions of Accuracy and F1-score for the four methods largely overlap, indicating that their classification performance is generally comparable. RRA exhibits slightly higher box positions and mean values, together with a relatively smaller dispersion across datasets, suggesting a certain degree of robustness. Furthermore, a non-parametric Friedman test [32] was conducted to statistically compare the Accuracy and F1-score of the four methods across the six datasets. The resulting *p*-values were approximately 0.49 for Accuracy and 0.28 for F1-score, both greater than 0.05, indicating that the null hypothesis of “no performance difference among the methods” cannot be rejected. Taken together with the graphical results, these findings suggest that RRA does not exhibit a statistically significant disadvantage in classification performance compared with the other aggregation strategies and in fact achieves slightly better Accuracy and F1-score on most datasets, implying that RRA is at least non-inferior—and in some cases advantageous—while not compromising Classification Accuracy.

Taken together, these results indicate that RRA achieves the highest stability scores on most datasets and across different Top-K settings, while maintaining classification performance in terms of Accuracy and F1-score that is overall comparable to that of the other aggregation strategies. This suggests that RRA can serve as a more robust and consistent rank aggregation module within the filter-ensemble component. Based on this finding, RRA is adopted as the fixed rank aggregation strategy in all subsequent experiments, and different feature selection and modeling methods are coupled with the RRA-generated candidate feature space to comparatively analyze the performance of the overall framework.

### 4.2. Comparison of Embedded Sparse Modeling Strategies Within the RRA Candidate Space

**Figure 4:**
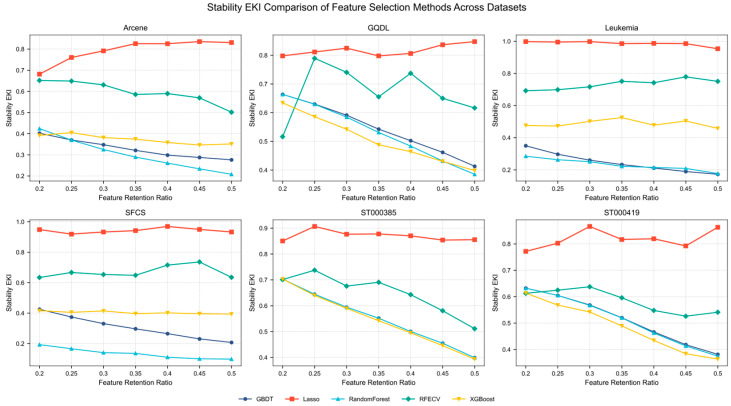
Comparison of EKI across datasets for different feature selection frameworks.

Previous results have shown that adopting RRA as the rank aggregation strategy can markedly improve feature selection stability without sacrificing classification performance. Building on this conclusion, RRA is fixed in this section as the filter-ensemble component, and multiple embedded and wrapper methods—including Lasso, GBDT, Random Forest, RFECV, and XGBoost—are coupled with it as refinement modules within the candidate feature space generated by RRA. In this way, five synergistic RRA-based feature selection frameworks are constructed, namely RRA+GBDT, RRA+Lasso, RRA+Random Forest, RRA+RFECV, and RRA+XGBoost, where the labels “GBDT”, “Lasso”, etc., in the figures all refer to the corresponding RRA-based frameworks, with RRA providing the heterogeneous filter ensemble and the specified method serving as the refinement model. Subsequently, the performance of these combinations on the six omics datasets is systematically compared from three perspectives: the stability metric EKI, classification performance (Accuracy and F1-score), and feature compression capability, in order to assess the overall advantages of FRL-TSFS at the framework level. Figure 4 presents the EKI-based stability comparison of the different combinations across all datasets.

In terms of the stability metric EKI, FRL-TSFS exhibits the highest overall feature selection stability on all six datasets, with a mean EKI value of 0.871, which is markedly higher than that of the second-best combination, RRA+RFECV (0.651), corresponding to a relative improvement of approximately 33.8%. In contrast, tree-based refinement strategies (RRA+XGBoost, RRA+GBDT, and RRA+RF) show substantially weaker overall stability, with mean EKI values only in the range of 0.37–0.47. Notably, on the typical high-dimensional, small-sample datasets Leukemia and SFCS, the advantage of FRL-TSFS becomes even more pronounced, with EKI values of 0.986 and 0.942, respectively. These results indicate that under a 10% data perturbation scenario, the framework can consistently produce highly overlapping feature subsets, which is beneficial for improving the reproducibility and biological interpretability of biomarker selection results. Overall, the findings suggest that, compared with tree-based models that are more susceptible to feature redundancy and random fluctuations, the L1-regularized sparse modeling mechanism of Lasso is better suited for robust feature extraction from high-dimensional omics data, and its combination with heterogeneous RRA-based ensemble filtering further amplifies this stability advantage.

**Figure 5:**
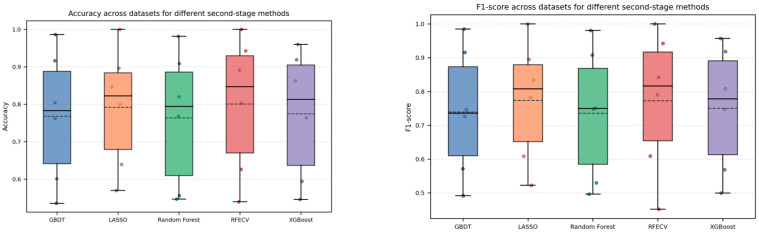
Boxplots of Accuracy and F1-score for RRA-based feature selection frameworks.

In this subsection, we further examine the classification performance of the FRL-TSFS framework. Specifically, on the six omics datasets, we compute the Accuracy and F1-score for five RRA-based feature selection methods, and the detailed results are summarized in Supplementary Information Tables S4 and S5. Based on these results, boxplots are drawn in Figure 5, where scatter points for each dataset are overlaid on the boxes to illustrate the overall performance distribution and variability of each method across datasets. In addition, a Friedman test is applied to the Accuracy and F1-score of the five frameworks over the six datasets to assess whether the observed differences in classification performance are statistically significant.

From Figure 5, it can be observed that the box heights and scatter distributions of Accuracy and F1-score for the five RRA-based feature selection frameworks largely overlap, indicating that their overall classification performance is broadly comparable. Except for relatively difficult datasets such as GQDL and ST000419, the Accuracy on most datasets exceeds 0.8, and on high-dimensional, small-sample datasets such as Leukemia and SFCS, all methods achieve classification accuracies close to 1.0. Overall, the frameworks based on Lasso and RFECV show a slight advantage on several datasets: Lasso attains higher Accuracy and F1-scores on datasets such as GQDL and ST000419, whereas RFECV performs marginally better on Arcene, Leukemia, SFCS, and ST000385; however, the differences among the methods remain generally small. This observation is further supported by the Friedman test results: the test statistics for Accuracy and F1-score are approximately 8.91 and 8.77, with corresponding *p*-values of 0.063 and 0.067, both greater than 0.05 and thus not reaching statistical significance. Therefore, it can be concluded that, on the six omics datasets considered, there is no statistically significant difference in classification Accuracy among the five RRA-based feature selection frameworks. Combined with the preceding stability analysis, these findings indicate that the proposed FRL-TSFS framework substantially improves the stability of feature selection while maintaining classification performance that is not inferior to the other frameworks, suggesting that its main advantage lies in enhancing the consistency and reproducibility of biomarker selection without compromising classification effectiveness.

**Figure 6:**
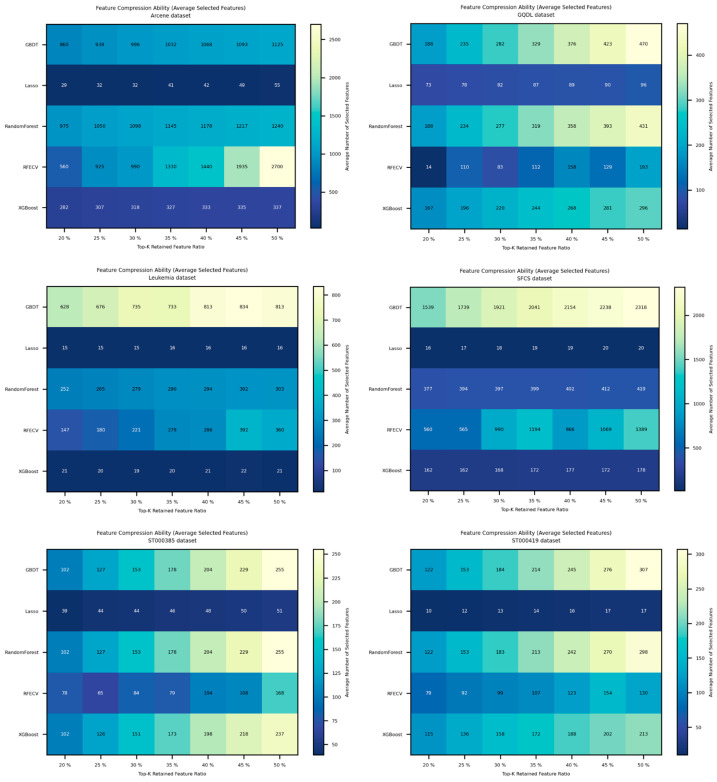
Heatmap comparing feature compression across different RRA-based feature selection frameworks.

In untargeted metabolomics, selecting as few yet representative biomarkers as possible not only reduces downstream experimental validation costs but also enhances model interpretability and clinical translational potential. Therefore, beyond stability and classification accuracy, a method’s feature compression capability is also a key criterion of merit. In this section, we systematically evaluate the feature compression capability of five RRA-based feature selection frameworks. Figure 6 reports their average control over the number of selected features across six omics datasets. The horizontal axis denotes the retained top-ranked feature proportion according to the RRA ranking, and the vertical axis gives the average number of finally selected features under 10-fold cross-validation.

From the results in Figure 6, it can be seen that when Lasso is coupled with the RRA-based candidate space (i.e., the FRL-TSFS framework), it consistently selects the smallest number of features across all retained feature proportions, exhibiting the strongest feature compression capability. For example, on typical high-dimensional, small-sample datasets such as Arcene, Leukemia, and SFCS, where the original dimensionality exceeds 7000 features, when RRA retains the top 50% of candidate features, FRL-TSFS ultimately selects only about 50, 16, and 20 features on Arcene, Leukemia, and SFCS, respectively. In contrast, tree-based combinations such as RRA+GBDT and RRA+Random Forest often retain several hundred to over a thousand features, while RFECV selects as many as 1388 features on SFCS, far exceeding the level achieved by Lasso. A similar pattern is observed on datasets such as GQDL, ST000385, and ST000419: across all retained feature proportions, the number of features selected by the Lasso-based combination remains the lowest among all methods, indicating that FRL-TSFS can achieve much stronger dimensionality reduction while maintaining classification performance.

As the retained feature proportion increases from 20% to 50%, the number of selected features rises for all methods, but the growth rate of FRL-TSFS is markedly slower than that of the other approaches and is almost flat on some datasets. For instance, on the ST000419 dataset, when the retained proportion increases from 20% to 50%, the number of selected features for GBDT grows from 122 to 307, and for Random Forest from 121.9 to 298.2, whereas the Lasso-based combination increases only from 9.9 to 16.9 features, resulting in a much smoother and more controlled growth curve. This suggests that Lasso can effectively suppress the accumulation of redundant features: even when a large number of candidates are retained in the initial screening step, the L1 regularization–induced sparsity constraint can still shrink unimportant or highly correlated variables, yielding a compact feature subset. By contrast, methods such as RFECV and Random Forest tend to select excessively large feature sets on multiple datasets, with limited dimensionality reduction, which reduces the interpretability of the results and, to some extent, limits their applicability in high-dimensional, small-sample metabolomics settings.

To quantify redundancy among the selected features, we computed pairwise Pearson correlation coefficients on the normalized data matrix for both the RRA-defined candidate subsets (Top-30% features) and the final L1-selected subsets. For each subset, we summarized the redundancy level using the mean absolute correlation |*r*| and the proportion of feature pairs with |**r**| > 0.8. These statistics were reported for all six datasets (Table 2) to assess whether the embedded L1 regularization actually reduces the correlation structure compared with the pre-filtered candidate sets.

For the RRA Top-30% candidate subsets, the mean absolute pairwise correlation |**r**| ranged from 0.24 to 0.44 (average 0.34), whereas for the L1-selected subsets it decreased to 0.19–0.27 (average 0.22). Similarly, the proportion of feature pairs with |**r**| > 0.8 dropped from an average of approximately 4.3% in the RRA candidate sets to about 1.4% in the L1-selected sets. For example, on the Arcene dataset, the RRA Top-30% candidate subset contained 3000 features with a mean |**r**| of 0.4372 and 8.2% of feature pairs showing |**r**| > 0.8, whereas the FRL-TSFS subset contained only 28 features with a reduced mean |**r**| of 0.2646 and 3.7% of highly correlated pairs. On the Leukemia dataset, the mean |r| decreased from 0.3826 to 0.1872 and the proportion of pairs with |**r**| > 0.8 from 0.95% to 0.03% when moving from the RRA candidate set (2138 features) to the L1-selected subset (15 features). Comparable trends were observed for the remaining datasets, indicating that FRL-TSFS not only compresses the feature space to a small number of metabolites or genes, but also effectively suppresses highly redundant features and yields more compact, less correlated feature subsets.

Finally, to examine whether the feature panels obtained by the embedded sparse models are biologically meaningful, we compared the FRL-TSFS feature panels with the results of conventional univariate differential analysis applied to all features. As summarized in Table 3, on the two metabolomics datasets ST000385 and ST000419, 43 out of 44 metabolites (97.7%) and 9 out of 12 metabolites (75.0%), respectively, in the FRL-TSFS panels were also identified as significantly differentially abundant (FDR < 0.05). Similar trends were observed on most of the remaining datasets: 25 of 34 features (73.5%) on Arcene, 44 of 80 features (55.0%) on GQDL, and all 15 features (100%) on Leukemia were classified as DE features, whereas only 2 of 18 features (11.1%) in the SFCS panel passed the FDR threshold. These findings indicate that a substantial proportion of the features highlighted by the stability-oriented FRL-TSFS framework are supported by conventional single-feature differential analysis, while the remaining features may mainly contribute through multivariate interactions, suggesting that the resulting panels are not only compact and stable but also biologically meaningful.

### 4.3. Ablation Study of Embedded Methods Without the RRA Stage

In addition, to evaluate the actual contribution of the RRA-based heterogeneous ensemble ranking module to the overall stability, a comparison setting was designed in which the filter-based heterogeneous integration is removed and only the embedded feature selectors are retained. Specifically, without introducing the RRA-based rank aggregation module, embedded methods such as Lasso, Random Forest, and GBDT are directly applied to the full feature space for feature selection, and the EKI as well as classification performance metrics are computed under the same 10-fold cross-validation perturbation scenario. By comparing these results with those of the full FRL-TSFS framework, the independent contribution of the RRA-based filter-ensemble ranking module to improving feature selection stability and feature compression capability can be analyzed from an ablation study perspective.

In the ablation experiments, the results for EKI, Accuracy, and F1-score are summarized in Table 4, Table 5 and Table 6. Overall, when the RRA module is removed and the embedded methods are directly applied to the full feature space, the stability of feature selection is clearly inferior to that of the full FRL-TSFS framework. On the one hand, in terms of the EKI metric, Lasso achieves an EKI of approximately 0.6336 on the Arcene dataset, which is substantially higher than those of GBDT (0.1595), RF (0.0834), and XGBoost (0.2644), yet still noticeably lower than the stability level attained by FRL-TSFS on the same dataset in the previous experiments. On the Leukemia dataset, the EKI of Lasso reaches 0.7506, whereas GBDT and RF attain only 0.0484 and 0.121, respectively, and XGBoost reaches merely 0.5268, indicating that tree-based ensemble methods are extremely sensitive to sample perturbations under high-dimensional, small-sample conditions. The situation is even more striking on datasets such as SFCS, ST000385, and ST000419: aside from Lasso (with EKIs of 0.4605, 0.6528, and 0.5767, respectively), most embedded methods yield EKI values that are almost close to zero. For example, the EKI of RF on ST000385 is only 0.0007, and the EKI of GBDT and SVM-RFE remains below 0.04 on most omics datasets, suggesting that the selected feature subsets are virtually non-reproducible under mild perturbations. Although SVM-RFE attains a relatively high EKI of 0.9116 on Arcene, its EKI values on the other five datasets fall between 0.0023 and 0.0044, which is close to random, reflecting a strong sensitivity to specific data structures and a lack of cross-dataset stability.

On the other hand, with respect to classification performance, when the heterogeneous RRA-based aggregation step is omitted, the embedded methods exhibit broadly comparable Accuracy and F1-score. On datasets such as Arcene and Leukemia, most methods achieve Accuracy and F1-score in the ranges of 0.80–0.91 and 0.79–0.99, respectively. On GQDL, ST000385, and ST000419, the differences in Accuracy among Lasso, RF, RFECV, and XGBoost are generally within about 0.05–0.08, and the fluctuations in F1-score are also limited. Combining the EKI and classification results, it can be seen that when relying solely on embedded feature selectors, even though acceptable Accuracy and F1-score can be obtained on some datasets, the corresponding feature subsets are highly unstable across different cross-validation folds, and it is difficult to achieve the same level of feature compression as FRL-TSFS while maintaining discriminative performance. In contrast, the integrated design of FRL-TSFS—first constructing a robust candidate ranking via filter-based heterogeneous ensemble and RRA, then performing L1-based sparse refinement within the candidate space—substantially enhances feature selection stability under comparable classification performance and compresses the final feature set to the scale of only several tens of metabolites or genes. This indicates that the RRA-based heterogeneous rank aggregation is not a redundant component but, together with L1-sparse modeling, constitutes a key source of the stability advantage of FRL-TSFS.

Therefore, the ablation experiments provide negative evidence supporting the necessity of the proposed “heterogeneous filter ensemble + L1-sparse modeling” synergy. The observed stability gains do not arise from simply stacking multiple embedded models, nor can they be attributed to any single method alone. Rather, only when task-aware L1-sparse modeling is applied within the robust candidate space pre-screened by RRA can a more balanced trade-off among stability, feature compression, and classification performance be achieved.

### 4.4. Sensitivity Analysis of the Penalty Parameter C

To investigate whether the conclusions of this study depend critically on the choice of the regularization strength in the embedded sparse model, we further examined the effect of varying the penalty parameter C in the L1-regularized logistic regression. For each dataset, C was swept over the grid {0.3, 0.5, 1.0, 2.0, 3.0}, and the corresponding stability (EKI), classification Accuracy, F1-score, and number of selected features were recorded. The detailed results are summarized in Supplementary Tables S6–S9 (EKI in Table S6, Accuracy in Table S7, F1-score in Table S8, and the number of selected features in Table S9).

Overall, changing C within this range led to only moderate variations in sparsity, stability, and predictive performance. Smaller C values generally produced more aggressive feature shrinkage and slightly higher EKI at the cost of minor decreases in Accuracy, whereas larger C values resulted in less shrinkage and mildly reduced stability. Importantly, across all tested values of C, the proposed FRL-TSFS framework consistently maintained higher stability and stronger feature compression than the competing methods, indicating that the main conclusions are robust with respect to the choice of C.

### 4.5. Comparison with Subsampling-Based Lasso Stability Selection

To further situate FRL-TSFS among existing stability-oriented methods, we implemented a subsampling-based Lasso stability selection baseline (SS-Lasso) following the general strategy of Meinshausen and Bühlmann [11]. For each training fold, multiple subsamples were drawn from the training data, L1-regularized logistic regression models were fitted on each subsample, and selection frequencies were computed for all features. Features whose frequencies exceeded a predefined threshold were retained, and a linear SVM classifier was then trained and evaluated under the same 10-fold cross-validation protocol as used for FRL-TSFS. Feature-selection stability (EKI), mean Accuracy, mean F1-score, and the number of selected features were computed in exactly the same way as for FRL-TSFS. For a fair comparison, FRL-TSFS was evaluated under its default configuration, in which the top-25% of features according to the RRA consensus ranking were used as the candidate set and the embedded L1-regularized logistic regression employed a penalty parameter of C = 0.5.

The quantitative comparison is summarized in Table 7. Across all six datasets, FRL-TSFS consistently achieves higher EKI values than SS-Lasso, indicating more stable feature subsets under the unified “10% data perturbation” scenario. In addition, FRL-TSFS attains equal or better classification performance: both Accuracy and F1-score are higher on most datasets, with particularly marked gains on SFCS, ST000385, and ST000419. In terms of sparsity, both approaches yield compact panels whose sizes range from 6 to 39 features, which is negligible compared with the original dimensionality (hundreds to more than ten thousand variables). FRL-TSFS tends to retain slightly more features than SS-Lasso in exchange for the observed gains in stability and predictive performance, but the resulting panels remain small in absolute terms. At the same time, FRL-TSFS avoids the substantial computational overhead incurred by repeated subsampling and refitting in SS-Lasso, providing a more efficient yet highly stable alternative for biomarker-oriented feature selection.

## 5. Discussion

The experimental results of this study consistently demonstrate, from a unified perspective, the effectiveness of a stability-first heterogeneous ensemble feature selection framework. Analyses based on the Extended Kuncheva Index (EKI) show that, under the mildly perturbed scenario induced by 10-fold cross-validation, the feature subsets obtained by FRL-TSFS exhibit much higher consistency across different sample partitions. This indicates that heterogeneous filter-based scoring combined with Robust Rank Aggregation can effectively suppress the bias and random fluctuations of individual methods. Within the candidate space constructed by RRA, the Lasso component yields sparse solutions with relatively stable convergence paths through L1 regularization, thereby compressing redundant features while preserving the reproducibility of the selected subsets. On most datasets, FRL-TSFS substantially outperforms other feature selection combinations in terms of EKI, while exhibiting only negligible differences in Accuracy and F1-score. These findings suggest that, in high-dimensional, small-sample metabolomics settings, stability and feature compression can be treated as optimization objectives of equal importance to discriminative performance, without incurring a meaningful loss in Classification Accuracy. In addition, the comparison among different embedded and ensemble baselines also indicates that nonlinear tree-based models that do not explicitly account for stability constraints tend to be highly sensitive to sample perturbations in small-sample regimes.

The experiments further reveal a certain coupling between feature selection stability and model performance. Methods with higher stability are more likely to maintain consistent and reliable predictive performance across multiple data splits, which helps reduce performance fluctuations and overfitting risks induced by sample perturbations. This underscores the positive role of improving feature selection consistency in enhancing the generalization ability of downstream predictive tasks. By contrast, some tree-based frameworks (such as GBDT and RF) are more sensitive to data perturbations in small-sample scenarios, leading to large fluctuations in the selected features and overall stability and classification performance that are less satisfactory than those of FRL-TSFS.

In this context, the rank-based design of the first stage in FRL-TSFS plays a specific and limited role. The five filter methods produce importance scores on very different numerical scales and with different distributional properties, which makes it difficult to aggregate raw scores in a fair way. By mapping these scores to ranks before applying RRA, the aggregation step becomes invariant to monotonic transformations and scale differences and less sensitive to outliers or heavy-tailed score distributions of any single filter. This facilitates a balanced integration of heterogeneous scoring criteria and contributes to the observed gains in stability. At the same time, we deliberately restrict the use of ranks to the feature selection stage: both the embedded L1-regularized logistic regression model and the final linear SVM classifier are trained on normalized measurement values rather than rank-transformed features. In this way, FRL-TSFS benefits from the robustness of rank-based aggregation for selection while preserving information on absolute effect sizes and class-dependent abundance differences that may be important for predictive performance and biological interpretation at the modeling stage.

Despite these encouraging results, several limitations of the present study should be acknowledged. First, our evaluation mainly focuses on within-dataset stability under a mildly perturbed scenario induced by 10-fold cross-validation, rather than on cross-study stability across independent cohorts. Although we considered six publicly available metabolomics and gene expression datasets to demonstrate the general applicability of FRL-TSFS, these datasets correspond to different diseases, platforms, and preprocessing pipelines, and therefore do not form matched training–validation pairs for the same biological question. A rigorous external validation of biomarker panels would require multiple independent cohorts that share comparable experimental protocols and feature spaces, together with additional steps for batch-effect correction and domain adaptation, which goes beyond the scope of this methodological work. In future studies, we plan to assemble multi-cohort datasets for the same disease or clinical endpoint and to systematically investigate the cross-dataset robustness of FRL-TSFS, as well as its extension to joint feature selection across harmonized multi-cohort data.

## 6. Conclusions

This study addresses the key problem of unstable biomarker selection in high-dimensional, small-sample omics data and proposes a biomarker stability-oriented framework, FRL-TSFS, that is synergistically driven by Robust Rank Aggregation and L1-sparse modeling. By aggregating feature rankings from multiple filter-based methods via RRA and combining them with the sparse regularization mechanism of Lasso, the framework achieves a favorable balance among feature selection stability, classification performance, and feature compression capability. The overall experimental results demonstrate that FRL-TSFS can substantially improve the consistency of selected features and markedly reduce the final number of features, while maintaining classification Accuracy and F1-score at levels comparable to competing methods. As a result, FRL-TSFS yields compact and reproducible biomarker panels that exhibit low redundancy and strong class-dependent abundance differences, particularly in the metabolomics datasets (Table 2 and Table 3). These properties support the biological interpretability of the resulting panels and provide a practical solution for stability-oriented feature selection and biomarker discovery in untargeted metabolomics and other omics studies under small-sample, high-noise conditions.

Future work will further investigate the cross-dataset robustness of FRL-TSFS on harmonized multi-cohort data and extend the framework to more complex scenarios, such as multi-omics data integration, multi-task learning, and the combination with automated feature screening and interpretable modeling techniques, with the ultimate goal of providing more stable and biologically meaningful support for precision medicine and bioinformatics-driven knowledge discovery.

## Figures and Tables

**Table 1:** Basic characteristics of the six metabolomics/omics datasets.

Dataset Name	Number of Features	Number of Samples	Number of Classes	Feature Type
Arcene	10,000	200	2	Genes
GQDL	940	78	5	Metabolites
Leukemia	7129	72	2	Genes
SFCS	10,283	54	3	Metabolites
ST000419	614	80	4	Metabolites
ST000385	511	181	7	Metabolites

**Table 2:** Redundancy analysis of RRA Top-30% candidate subsets and L1-selected subsets.

	RRA Top-30%	Mean |r|	Proportion of Pairs with |r| > 0.8	L1-Selected Subset	Mean |r|	Proportion of Pairs with |r| > 0.8
Arcene	3000	0.4372	0.0819	28	0.2646	0.0370
GQDL	282	0.3676	0.0569	79	0.2222	0.0087
Leukemia	2138	0.3826	0.0095	15	0.1872	0.0003
SFCS	3084	0.3202	0.0196	18	0.2189	0.0089
ST000385	153	0.2439	0.0274	44	0.1905	0.0135
ST000419	184	0.3164	0.0625	12	0.2367	0.0151

**Table 3:** Overlap between FRL-TSFS feature panels and significantly differentially expressed/abundant (DE) features (FDR < 0.05) across the six datasets.

	No. of Features in FRL-TSFS Panel	No. of Panel Features Evaluated in Differential Analysis	No. of DE Features in Panel (FDR < 0.05)	Proportion of DE Features in Panel
Arcene	34	34	25	0.7353
GQDL	80	80	44	0.5500
Leukemia	15	15	15	1.0000
SFCS	18	18	4	0.2222
ST000385	44	44	43	0.9773
ST000419	12	12	9	0.7500

**Table 4:** Comparison of Stability (EKI) of Various Embedded and Wrapper-Based Feature Selection Methods Without RRA on Different Datasets.

	Arcene	GQDL	Leukemia	SFCS	ST000385	ST000419
GBDT	0.160	0.041	0.048	0.043	0.042	0.025
Lasso	0.634	0.659	0.751	0.461	0.653	0.577
Random Forest	0.083	0.043	0.121	0.064	0.001	0.044
RFECV	0.912	0.003	0.002	0.002	0.004	0.002
XGBoost	0.264	0.231	0.527	0.198	0.185	0.209

**Table 5:** Comparison of Accuracy of Various Embedded and Wrapper-Based Feature Selection Methods Without RRA on Different Datasets.

	Arcene	GQDL	Leukemia	SFCS	ST000385	ST000419
GBDT	0.855	0.538	0.943	0.426	0.779	0.498
Lasso	0.800	0.489	0.943	0.444	0.746	0.501
Random Forest	0.895	0.563	0.971	0.537	0.806	0.465
RFECV	0.910	0.564	0.986	0.370	0.780	0.501
XGBoost	0.835	0.532	0.957	0.556	0.745	0.473

**Table 6:** Comparison of F1-score of Various Embedded and Wrapper-Based Feature Selection Methods Without RRA on Different Datasets.

	Arcene	GQDL	Leukemia	SFCS	ST000385	ST000419
GBDT	0.855	0.496	0.942	0.328	0.756	0.486
Lasso	0.797	0.462	0.944	0.369	0.740	0.467
Random Forest	0.895	0.507	0.970	0.457	0.799	0.458
RFECV	0.910	0.524	0.985	0.315	0.756	0.493
XGBoost	0.832	0.513	0.955	0.472	0.726	0.462

**Table 7:** Comparison of FRL-TSFS and SS-Lasso on six omics datasets(Feature-selection stability (Extended Kuncheva Index, EKI), mean Accuracy, mean F1-score, and the number of selected features are reported for each method).

		Arcene	GQDL	Leukemia	SFCS	ST000385	ST000419
EKI	SS- Lasso	0.5545	0.6846	0.5638	0.2799	0.6803	0.5600
Accuracy		0.7400	0.4982	0.9036	0.6852	0.7128	0.4148
F1		0.7356	0.4298	0.8979	0.6746	0.7069	0.4029
The number of features		18.0	73.0	6.0	7.0	26.0	8.0
EKI	FRL- TSFS	0.6813	0.7976	0.9978	0.9484	0.8501	0.7714
Accuracy		0.875	0.5625	1.000	0.8148	0.7678	0.6126
F1		0.8728	0.5132	1.000	0.8048	0.7579	0.5598
The number of features		29.0	72.0	15.0	15.0	39.0	10.0

## Data Availability

The data presented in this study are available on reasonable request from the corresponding author. The data are not publicly available due to privacy/ethical restrictions.

## Supplementary Materials

The following supporting information can be downloaded at: https://www.mdpi.com/article/10.3390/metabo15120806/s1, the Supplementary Materials include Tables S1–S9, providing detailed classification performance and additional experimental results.
