# Supplementary material for: A Stability-Oriented Biomarker Selection Framework Synergistically Driven by Robust Rank Aggregation and L1-Sparse Modeling
Source: Metabolites. 2025 Dec 18;15(12):806. doi: 10.3390/metabo15120806 (PMC12735098; doi:10.3390/metabo15120806)
Supplement: Supplementary file 1 [file metabolites-15-00806-s001.zip › metabolites-4033385-supplementary.pdf]

SUPPLEMENT

A Stability-Oriented Biomarker Selection Framework Synergistically Driven by Robust Rank Aggregation and L1-Sparse Modeling

Jigen Luo [1,2], Jianqiang Du [3,*], Jia He [1,2], Qiang Huang [1,2], Zixuan Liu [1,2] and Gaoxiang Huang [1,2]

1 School of Intelligent Medicine and Information Engineering, Jiangxi University of Chinese Medicine, Nanchang 330004, China; 20192002@jxutcm.edu.cn (J.L.); 20201079@jxutcm.edu.cn (J.H.); 20201041@jxutcm.edu.cn (Q.H.); liuzixuan@jxutcm.edu.cn (Z.L.); huanggaoxiang@jxutcm.edu.cn (G.H.)
2 Key Laboratory of Traditional Chinese Medicine Artificial Intelligence of Jiangxi Province, Nanchang 330004, China
3 School of Mathematics and Information Science, Nanchang Normal University, Nanchang 330032, China
* Correspondence: jianqiang_du@163.com; Tel.: +86-18170069045

Section S1: Table

Before feature selection and model training, we benchmarked several normalization schemes across the six datasets, as described in Section 3.2.1. Table S1 summarizes the results of this comparison. Overall, level scaling showed the most favorable variability profile among the tested schemes and was therefore adopted as the default normalization strategy for the gene expression datasets in the main analysis.

To evaluate the classification performance of RRA relative to the other three aggregation strategies, we computed the mean Accuracy and F1-score under 10-fold cross-validation at different Top-K settings, that is, when retaining the top K ranked features in each case. The numerical results are reported in Supplementary Tables S2 and S3.

In addition, to assess the classification performance of different RRA-based feature selection frameworks under varying levels of feature retention, we considered a series of top-ranked feature proportions according to the RRA ordering (20 %, 25 %, 30 %, 35 %, 40 %, 45 %, and 50 %), passed the corresponding candidate subsets to the downstream models, and computed the mean Accuracy and F1-score under 10-fold cross-validation. These results are summarized in Supplementary Tables S4 and S5.

Finally, to examine whether the conclusions of this study depend critically on the choice of the regularization strength in the embedded sparse model, we conducted a sensitivity analysis of the penalty parameter C in the L1-regularized logistic regression. For each dataset, C was varied over the grid {0.3, 0.5, 1.0, 2.0, 3.0}, and the corresponding feature selection stability (Extended Kuncheva Index, EKI), classification Accuracy, F1-score, and number of selected features were evaluated for the proposed FRL-TSFS framework and the other L1-based baselines. The detailed numerical results of this analysis are reported in Supplementary Tables S6 – S9, where S6 summarizes the EKI values, S7 reports the Accuracy, S8 reports the F1-scores, and S9 summarizes the numbers of selected features under each value of C.

Table S1. omparison of normalization schemes across the six benchmark datasets.

	Arcene			GQDL			Leukemia		
	**PCV**	**PEV**	**PMAD**	**PCV**	**PEV**	**PMAD**	**PCV**	**PEV**	**PMAD**

Raw Data	1.0991	6101.14	30.1207	0.1395	1.5880	0.4757	1.3954	747763	205.57
AutoScaling	12755.13	0.9751	0.3244	674.57	0.8320	0.4449	17181	0.9798	0.5491
VSN	-0.9696	3728.08	1.5961	0.0012	0.0001	0.0042	0.1392	1.0193	0.6761
LogTransform	0.7248	2.7648	0.5419	0.1253	0.1253	0.0781	1.0237	0.7315	0.4219
LevelScaling	1.1177	0.0424	0.0778	0.4592	0.3108	0.0857	0.4592	0.0351	0.1048
CyclicLoess	0.7571	4854.64	28.4896	0.1387	1.4829	0.5227	1.0801	655839	211.25

	**SFCS**			**ST000385**			**ST000419**		
	**PCV**	**PEV**	**PMAD**	**PCV**	**PEV**	**PMAD**	**PCV**	**PEV**	**PMAD**
Raw Data	2.2643	4.25*10^7	441.76	2.5117	9.58*10^7	905.73	3.7978	1.32*10^9	2423.08
AutoScaling	1.43*10^17	0.9134	0.4431	7685.3	0.8821	0.3926	586.47	0.9427	0.3816
VSN	0.1004	0.5654	0.3692	0.0976	0.7886	0.4765	0.1098	1.1761	0.5404
LogTransform	0.1866	0.8793	0.3422	0.1218	0.5758	0.3849	0.1287	0.7586	0.4151
LevelScaling	0.6342	0.0436	0.0975	0.7069	0.0237	0.0669	0.8228	0.0442	0.0806
CyclicLoess	1.6738	2.9*10^7	497.64	1.7341	7.49*10^7	1066.49	1.8961	4.49*10^8	4394.75

Table S2. Comparison of Accuracy of Different Aggregation Strategies on Various Datasets

**Data**	**Aggregation strategy**	**Top-10**	**Top-20**	**Top-30**	**Top-40**	**Top-50**	**Top-60**	**Top-70**	**Top-80**	**Top-90**	**Top-100**
Arcene		0.730	0.645	0.690	0.665	0.745	0.800	0.810	0.805	0.765	0.780
GQDL		0.502	0.529	0.618	0.575	0.579	0.539	0.550	0.618	0.482	0.588
Leukemia	Borda	0.902	0.929	0.945	0.959	0.971	0.945	0.959	0.943	0.986	0.971
SFCS		0.796	0.778	0.907	0.889	0.796	0.907	0.815	0.870	0.870	0.796
ST000385		0.437	0.602	0.630	0.674	0.686	0.729	0.712	0.740	0.746	0.768
ST000419		0.324	0.513	0.501	0.596	0.574	0.661	0.615	0.550	0.597	0.563
Arcene		0.665	0.665	0.690	0.695	0.635	0.675	0.670	0.690	0.660	0.670
GQDL		0.566	0.655	0.602	0.577	0.461	0.498	0.504	0.516	0.488	0.513
Leukemia	Mean	0.914	0.932	0.943	0.943	0.957	0.961	0.943	0.961	0.971	0.973
SFCS		0.778	0.833	0.796	0.852	0.833	0.833	0.852	0.815	0.815	0.889
ST000385		0.347	0.480	0.591	0.663	0.647	0.674	0.746	0.758	0.757	0.741
ST000419		0.436	0.361	0.448	0.437	0.597	0.723	0.591	0.660	0.674	0.688
Arcene		0.745	0.750	0.735	0.805	0.790	0.800	0.770	0.805	0.785	0.780
GQDL		0.539	0.516	0.486	0.564	0.604	0.589	0.509	0.473	0.521	0.523
Leukemia	RRA	0.945	0.929	0.945	0.945	0.943	0.943	0.930	0.957	0.957	0.959
SFCS		0.815	0.852	0.796	0.833	0.833	0.815	0.833	0.833	0.815	0.833
ST000385		0.618	0.630	0.625	0.669	0.652	0.674	0.714	0.696	0.712	0.724

ST000419		0.597	0.738	0.637	0.675	0.789	0.722	0.775	0.764	0.738	0.705
Arcene	Intersection	0.620	0.655	0.715	0.730	0.740	0.725	0.800	0.735	0.720	0.735
GQDL		0.425	0.530	0.518	0.513	0.500	0.584	0.513	0.580	0.523	0.598
Leukemia		0.943	0.943	0.943	0.971	0.986	0.971	0.986	0.957	0.973	0.986
SFCS		0.833	0.870	0.778	0.815	0.778	0.815	0.778	0.833	0.870	0.796
ST000385		0.602	0.586	0.619	0.679	0.696	0.696	0.713	0.751	0.735	0.768
ST000419		0.377	0.463	0.473	0.577	0.523	0.522	0.574	0.575	0.539	0.537

Table S3. Comparison of F1-score of Different Aggregation Strategies on Various Datasets

Data	Aggregation strategy	Top-10	Top-20	Top-30	Top-40	Top-50	Top-60	Top-70	Top-80	Top-90	Top-100
Arcene	Borda	0.727	0.634	0.687	0.654	0.743	0.799	0.810	0.805	0.763	0.778
GQDL		0.457	0.491	0.595	0.545	0.563	0.512	0.530	0.590	0.481	0.553
Leukemia		0.892	0.917	0.942	0.958	0.968	0.941	0.958	0.941	0.985	0.970
SFCS		0.758	0.752	0.883	0.859	0.769	0.897	0.795	0.848	0.833	0.766
ST000385		0.374	0.549	0.598	0.648	0.661	0.708	0.687	0.718	0.725	0.744
ST000419		0.224	0.454	0.464	0.542	0.537	0.635	0.592	0.515	0.569	0.540
Arcene	Mean	0.648	0.651	0.679	0.691	0.616	0.663	0.652	0.672	0.647	0.657
GQDL		0.555	0.614	0.573	0.545	0.414	0.479	0.485	0.495	0.470	0.504
Leukemia		0.909	0.929	0.941	0.939	0.955	0.957	0.940	0.960	0.969	0.971
SFCS		0.753	0.774	0.743	0.794	0.778	0.806	0.807	0.774	0.765	0.839
ST000385		0.268	0.411	0.560	0.634	0.616	0.645	0.730	0.743	0.736	0.723
ST000419		0.310	0.212	0.335	0.319	0.522	0.673	0.545	0.639	0.643	0.664
Arcene	RRA	0.742	0.748	0.730	0.804	0.788	0.799	0.765	0.804	0.783	0.779
GQDL		0.609	0.599	0.542	0.650	0.670	0.672	0.587	0.555	0.573	0.576
Leukemia		0.942	0.926	0.943	0.941	0.939	0.941	0.926	0.955	0.955	0.958
SFCS		0.743	0.785	0.724	0.783	0.759	0.764	0.780	0.769	0.758	0.769
ST000385		0.582	0.583	0.584	0.649	0.619	0.644	0.690	0.671	0.698	0.708
ST000419		0.559	0.682	0.607	0.600	0.739	0.671	0.743	0.729	0.683	0.671
Arcene	Intersection	0.609	0.628	0.715	0.728	0.739	0.724	0.799	0.732	0.716	0.732
GQDL		0.409	0.497	0.487	0.489	0.491	0.572	0.489	0.542	0.502	0.570
Leukemia		0.942	0.941	0.938	0.971	0.984	0.970	0.984	0.955	0.972	0.985
SFCS		0.803	0.848	0.766	0.796	0.768	0.798	0.763	0.807	0.838	0.751
ST000385		0.553	0.545	0.581	0.657	0.662	0.677	0.697	0.730	0.723	0.749
ST000419		0.352	0.403	0.447	0.523	0.482	0.494	0.536	0.542	0.509	0.503

Table S4. Comparison of Classification Accuracy between FRL-TSFS and other RRA-based feature selection frameworks across six omics datasets.

Data	Methods	20%	25%	30%	35%	40%	45%	50%
Arcene	GBDT	0.910	0.890	0.900	0.920	0.920	0.950	0.924
GQDL		0.5268	0.4982	0.5161	0.5107	0.5661	0.575	0.5554
Leukemia		0.9875	0.9875	0.9857	0.9857	0.9857	0.9857	0.9857
SFCS		0.7963	0.7963	0.7963	0.8148	0.8148	0.8148	0.7963
ST000385		0.7012	0.7573	0.7295	0.7898	0.7956	0.7898	0.7728
ST000419		0.6117	0.5733	0.6245	0.5998	0.6245	0.5504	0.6245
Arcene	Lasso	0.875	0.885	0.875	0.875	0.92	0.91	0.935
GQDL		0.5625	0.5393	0.5036	0.5786	0.5661	0.5911	0.6482
Leukemia		1.000	1.000	1.000	1.000	1.000	1.000	1.000
SFCS		0.8148	0.8889	0.8704	0.8333	0.8889	0.8148	0.8148
ST000385		0.7678	0.7904	0.7845	0.8123	0.8073	0.8175	0.8123
ST000419		0.6126	0.6163	0.6374	0.6474	0.6621	0.576	0.7234
Arcene	Random Forest	0.905	0.915	0.895	0.91	0.905	0.9	0.93
GQDL		0.5393	0.4696	0.5393	0.575	0.525	0.6161	0.5625
Leukemia		0.9857	0.9714	0.9857	0.9857	0.9732	0.9857	0.9857
SFCS		0.8148	0.8519	0.8333	0.8148	0.7778	0.8333	0.8148
ST000385		0.7023	0.7409	0.7839	0.7675	0.7904	0.7792	0.8184
ST000419		0.4872	0.587	0.5357	0.5504	0.5742	0.5495	0.6108
Arcene	RFECV	0.945	0.915	0.95	0.965	0.97	0.95	0.905
GQDL		0.6929	0.5107	0.5393	0.5071	0.55	0.5268	0.4482
Leukemia		1.000	1.000	1.000	1.000	1.000	1.000	1.000
SFCS		0.8889	0.8704	0.8889	0.9444	0.9259	0.8704	0.8519
ST000385		0.8284	0.7015	0.8129	0.8453	0.8342	0.7898	0.8061
ST000419		0.6374	0.6245	0.6593	0.6255	0.5879	0.6245	0.6245
Arcene	XGBoost	0.935	0.89	0.91	0.925	0.935	0.92	0.92
GQDL		0.5268	0.5554	0.5482	0.5018	0.4893	0.6429	0.5536
Leukemia		0.9571	0.9446	0.9571	0.9571	0.9714	0.9589	0.9732
SFCS		0.8704	0.8704	0.8333	0.8519	0.8889	0.8519	0.8704
ST000385		0.7234	0.7518	0.7295	0.7564	0.7962	0.7953	0.7956
ST000419		0.576	0.5623	0.5495	0.5852	0.6007	0.6502	0.6364

Table S5. Comparison of F1-Score between FRL-TSFS and other RRA-based feature selection frameworks across six omics datasets.

Data	Methods	20%	25%	30%	35%	40%	45%	50%
Arcene	GBDT	0.9095	0.89	0.8995	0.9199	0.9196	0.9496	0.9243
GQDL		0.4958	0.462	0.4874	0.4582	0.5019	0.5304	0.5082
Leukemia		0.9868	0.9868	0.984	0.984	0.984	0.984	0.9851
SFCS		0.7304	0.7167	0.7167	0.7327	0.7327	0.7327	0.723
ST000385		0.6821	0.7425	0.7102	0.7648	0.7892	0.782	0.7538
ST000419		0.5849	0.5408	0.6142	0.5685	0.5884	0.5128	0.5932
Arcene	Lasso	0.8728	0.8846	0.8746	0.8726	0.9192	0.9086	0.9342
GQDL		0.5132	0.5011	0.4502	0.5191	0.514	0.5587	0.6021
Leukemia		1.000	1.000	1.000	1.000	1.000	1.000	1.000
SFCS		0.8048	0.874	0.8641	0.8184	0.8709	0.8094	0.7969
ST000385		0.7579	0.7799	0.7575	0.7918	0.7951	0.8037	0.7935
ST000419		0.5598	0.5802	0.6122	0.6012	0.6371	0.5562	0.7141
Arcene	Random Forest	0.9045	0.9147	0.8936	0.9097	0.9044	0.8998	0.9296
GQDL		0.4965	0.4214	0.4737	0.532	0.4877	0.5419	0.5242
Leukemia		0.9851	0.9703	0.984	0.9851	0.9708	0.9851	0.9851
SFCS		0.7403	0.786	0.7787	0.7352	0.7082	0.7588	0.7327
ST000385		0.6808	0.7235	0.7736	0.7491	0.7585	0.7685	0.8059
ST000419		0.4488	0.5502	0.5164	0.5298	0.5515	0.5118	0.603
Arcene	RFECV	0.9446	0.9145	0.9498	0.9649	0.9699	0.9499	0.9053
GQDL		0.653	0.4504	0.3919	0.4463	0.5041	0.389	0.3303
Leukemia		1.000	1.000	1.000	1.000	1.000	1.000	1.000
SFCS		0.8381	0.811	0.8392	0.9185	0.8914	0.811	0.7849
ST000385		0.8133	0.6847	0.8035	0.8373	0.8312	0.7771	0.7901
ST000419		0.6273	0.6006	0.6363	0.5998	0.5812	0.607	0.6104
Arcene	XGBoost	0.9337	0.8895	0.9097	0.9248	0.9339	0.9188	0.9198
GQDL		0.475	0.5099	0.4856	0.4819	0.4535	0.5856	0.5081
Leukemia		0.9446	0.9443	0.9554	0.9571	0.9714	0.9557	0.9728
SFCS		0.812	0.8196	0.7764	0.8025	0.8457	0.786	0.821
ST000385		0.6948	0.727	0.7095	0.7501	0.7861	0.7867	0.7806
ST000419		0.5446	0.5352	0.4991	0.5616	0.5748	0.6422	0.6227

Table S6. Sensitivity of feature selection stability (EKI) to the penalty parameter C.

	**C-value**	**20%**	**25%**	**30%**	**35%**	**40%**	**45%**	**50%**
Arcene	0.3	0.8644	0.9176	0.8730	0.9268	0.9501	0.9245	0.9084
	0.5	0.6813	0.7601	0.7914	0.8254	0.8252	0.8352	0.8307
	1.0	0.5580	0.6285	0.6230	0.6545	0.6517	0.6599	0.6936
	2.0	0.5748	0.5650	0.5567	0.5385	0.5955	0.5662	0.6089
	3.0	0.5713	0.5715	0.5347	0.5330	0.5579	0.5822	0.5861
GQDL	0.3	0.8146	0.8238	0.8370	0.8285	0.8202	0.8444	0.8601
	0.5	0.7976	0.8112	0.8245	0.7976	0.8062	0.8365	0.8473
	1.0	0.7969	0.7879	0.7921	0.7794	0.7772	0.8172	0.8076
	2.0	0.7687	0.7700	0.7728	0.7632	0.7678	0.7882	0.7721
	3.0	0.7673	0.7631	0.7745	0.7623	0.7682	0.7692	0.7685
Leukemia	0.3	0.9985	0.9884	0.9823	0.9923	0.9984	0.9923	0.9871
	0.5	0.9979	0.9950	0.9978	0.9853	0.9869	0.9854	0.9535
	1.0	0.9712	0.9779	0.9870	0.9883	0.9847	0.9715	0.9827
	2.0	0.9690	0.9468	0.9555	0.9500	0.9564	0.9505	0.9452
	3.0	0.9681	0.9448	0.9386	0.9472	0.9351	0.9307	0.9259
SFCS	0.3	0.9992	0.9992	0.9992	0.9992	0.9992	0.9967	0.9944
	0.5	0.9485	0.9187	0.9326	0.9413	0.9690	0.9496	0.9326
	1.0	0.8317	0.8740	0.9174	0.9181	0.9414	0.9439	0.9489
	2.0	0.6992	0.7566	0.8034	0.8130	0.8683	0.8726	0.8884
	3.0	0.6914	0.7389	0.7850	0.7877	0.8265	0.8511	0.8568
ST000385	0.3	0.8970	0.9389	0.9006	0.9465	0.9459	0.9442	0.9505
	0.5	0.8501	0.9064	0.8764	0.8773	0.8700	0.8537	0.8551
	1.0	0.7728	0.7837	0.7401	0.7532	0.7362	0.7148	0.7253
	2.0	0.6979	0.7038	0.6833	0.6833	0.6544	0.6435	0.6402
	3.0	0.6815	0.6931	0.6539	0.6522	0.6212	0.6125	0.6136
ST000419	0.3	0.7919	0.8446	0.9280	0.9588	0.9884	0.9570	0.9397
	0.5	0.7715	0.8025	0.8659	0.8166	0.8193	0.7921	0.8629
	1.0	0.6328	0.6515	0.6995	0.6858	0.6906	0.7083	0.7529
	2.0	0.6206	0.6232	0.6433	0.6150	0.6180	0.6360	0.6950
	3.0	0.6140	0.6279	0.6316	0.6065	0.6116	0.6193	0.6450

Table S7. Sensitivity of classification Accuracy to the penalty parameter C.

	C-value	20%	25%	30%	35%	40%	45%	50%
Arcene	0.3	0.8150	0.8250	0.8350	0.8300	0.8450	0.8650	0.8650
	0.5	0.8750	0.8850	0.8750	0.8750	0.9200	0.9100	0.9350
	1.0	0.9000	0.9150	0.8900	0.9150	0.9050	0.9400	0.9400
	2.0	0.9450	0.9300	0.9300	0.9300	0.9300	0.9550	0.9550
	3.0	0.9450	0.9350	0.9000	0.9400	0.9650	0.9600	0.9550
GQDL	0.3	0.5768	0.6232	0.6286	0.5589	0.6679	0.6589	0.6893
	0.5	0.5625	0.5393	0.5036	0.5786	0.5661	0.5911	0.6482
	1.0	0.5500	0.6125	0.5911	0.6179	0.5911	0.5571	0.5696
	2.0	0.5411	0.5089	0.5250	0.6393	0.5786	0.5625	0.5839
	3.0	0.5768	0.5143	0.5214	0.6054	0.5661	0.5929	0.6321
Leukemia	0.3	0.9857	0.9750	0.9857	0.9857	0.9857	0.9857	0.9857
	0.5	1.0000	1.0000	1.0000	1.0000	1.0000	1.0000	1.0000
	1.0	1.0000	1.0000	1.0000	1.0000	1.0000	1.0000	1.0000
	2.0	1.0000	1.0000	1.0000	1.0000	1.0000	1.0000	1.0000
	3.0	1.0000	1.0000	1.0000	1.0000	1.0000	1.0000	1.0000
SFCS	0.3	0.7963	0.7778	0.7963	0.7593	0.7593	0.7593	0.7963
	0.5	0.8148	0.8889	0.8704	0.8333	0.8889	0.8148	0.8148
	1.0	0.9630	0.9259	0.9259	0.9259	0.9444	0.9630	0.9259
	2.0	0.9630	0.9815	0.9815	0.9815	0.9630	0.9815	0.9630
	3.0	0.9259	0.9444	0.9630	0.9630	0.9815	0.9630	0.9815
ST000385	0.3	0.7453	0.7137	0.7225	0.7184	0.7345	0.7406	0.7354
	0.5	0.7678	0.7904	0.7845	0.8123	0.8073	0.8175	0.8123
	1.0	0.7459	0.7848	0.8064	0.8231	0.8404	0.8173	0.8064
	2.0	0.7459	0.7515	0.8009	0.8018	0.8123	0.8012	0.8231
	3.0	0.7456	0.7734	0.7617	0.8287	0.8456	0.8231	0.8184
ST000419	0.3	0.5760	0.6639	0.5971	0.5522	0.6245	0.5989	0.4643
	0.5	0.6126	0.6163	0.6374	0.6474	0.6621	0.5760	0.7234
	1.0	0.6282	0.6392	0.6328	0.7234	0.7491	0.7381	0.7408
	2.0	0.7372	0.6154	0.6282	0.6731	0.6978	0.7491	0.6859
	3.0	0.6126	0.6108	0.6117	0.6868	0.6749	0.6731	0.6832

Table S8. Sensitivity of F1-score to the penalty parameter C.

	C-value	20%	25%	30%	35%	40%	45%	50%
Arcene	0.3	0.8101	0.8244	0.8324	0.8240	0.8421	0.8637	0.8643
	0.5	0.8728	0.8846	0.8746	0.8726	0.9192	0.9086	0.9342
	1.0	0.8995	0.9141	0.8895	0.9139	0.9033	0.9395	0.9396
	2.0	0.9448	0.9297	0.9291	0.9290	0.9299	0.9545	0.9545
	3.0	0.9450	0.9345	0.8991	0.9399	0.9639	0.9597	0.9546
GQDL	0.3	0.5297	0.5797	0.5780	0.5314	0.6294	0.6221	0.6632
	0.5	0.5132	0.5011	0.4502	0.5191	0.5140	0.5587	0.6021
	1.0	0.5117	0.5580	0.5412	0.5511	0.5449	0.5210	0.5077
	2.0	0.5013	0.4813	0.4834	0.5804	0.5474	0.5154	0.5519
	3.0	0.5176	0.4743	0.4954	0.5662	0.5049	0.5634	0.5631
Leukemia	0.3	0.9863	0.9745	0.9863	0.9863	0.9863	0.9857	0.9840
	0.5	1.0000	1.0000	1.0000	1.0000	1.0000	1.0000	1.0000
	1.0	1.0000	1.0000	1.0000	1.0000	1.0000	1.0000	1.0000
	2.0	1.0000	1.0000	1.0000	1.0000	1.0000	1.0000	1.0000
	3.0	1.0000	1.0000	1.0000	1.0000	1.0000	1.0000	1.0000
SFCS	0.3	0.7872	0.7792	0.7820	0.7640	0.7577	0.7694	0.7839
	0.5	0.8048	0.8740	0.8641	0.8184	0.8709	0.8094	0.7969
	1.0	0.9457	0.9035	0.8949	0.8949	0.9370	0.9468	0.9197
	2.0	0.9468	0.9728	0.9728	0.9728	0.9457	0.9728	0.9457
	3.0	0.8924	0.9196	0.9457	0.9468	0.9728	0.9457	0.9728
ST000385	0.3	0.7293	0.6975	0.6977	0.7020	0.7127	0.7249	0.7241
	0.5	0.7579	0.7799	0.7575	0.7918	0.7951	0.8037	0.7935
	1.0	0.7201	0.7702	0.7918	0.8156	0.8260	0.8043	0.7958
	2.0	0.7258	0.7370	0.7865	0.7884	0.7990	0.7870	0.8064
	3.0	0.7274	0.7572	0.7455	0.8087	0.8337	0.8008	0.8074
ST000419	0.3	0.5588	0.6570	0.5862	0.5264	0.6150	0.5939	0.4440
	0.5	0.5598	0.5802	0.6122	0.6012	0.6371	0.5562	0.7141
	1.0	0.6087	0.6240	0.6108	0.7045	0.7315	0.7252	0.7304
	2.0	0.7226	0.5854	0.6020	0.6575	0.6814	0.7240	0.6768
	3.0	0.5912	0.5710	0.5843	0.6742	0.6557	0.6600	0.6466

Table S9. Effect of the penalty parameter C on the number of selected features.

	C-value	20%	25%	30%	35%	40%	45%	50%
Arcene	0.3	22	23	26	27	28	30	31
	0.5	29	32	32	41	42	49	55
	1.0	43	48	56	66	77	81	87
	2.0	63	71	81	88	113	107	125
	3.0	84	97	111	115	116	137	148
GQDL	0.3	65	71	73	76	79	80	81
	0.5	73	79	82	87	89	90	96
	1.0	78	88	91	98	105	108	116
	2.0	88	98	105	112	123	123	135
	3.0	93	105	114	124	132	142	150
Leukemia	0.3	11	11	11	11	11	11	11
	0.5	15	15	15	16	16	16	16
	1.0	23	24	24	25	25	27	26
	2.0	29	30	31	32	33	34	34
	3.0	32	33	34	35	36	38	38
SFCS	0.3	8	8	8	8	8	8	9
	0.5	16	17	18	19	19	20	20
	1.0	36	38	39	42	44	45	48
	2.0	55	58	60	64	68	70	74
	3.0	66	69	73	79	85	90	94
ST000385	0.3	20	22	22	22	23	24	24
	0.5	39	44	44	46	48	50	51
	1.0	53	64	71	77	81	85	90
	2.0	68	80	90	95	100	106	111
	3.0	77	88	100	107	115	119	125
ST000419	0.3	5	5	6	6	7	7	8
	0.5	10	12	13	15	16	17	17
	1.0	25	29	34	37	38	40	40
	2.0	42	46	53	54	57	57	58
	3.0	48	56	62	63	65	66	68
